# Global SUMOylome Adjustments in Basal Defenses of *Arabidopsis thaliana* Involve Complex Interplay Between SMALL-UBIQUITIN LIKE MODIFIERs and the Negative Immune Regulator *SUPPRESSOR OF rps4-RLD1*

**DOI:** 10.3389/fcell.2021.680760

**Published:** 2021-09-30

**Authors:** Mritunjay Kasera, Kishor D. Ingole, Sakshi Rampuria, Yashika Walia, Walter Gassmann, Saikat Bhattacharjee

**Affiliations:** ^1^Laboratory of Signal Transduction and Plant Resistance, UNESCO-Regional Centre for Biotechnology (RCB), NCR Biotech Science Cluster, Faridabad, India; ^2^Kalinga Institute of Industrial Technology (KIIT) University, Bhubaneswar, India; ^3^Division of Plant Sciences, C. S. Bond Life Sciences Center and Interdisciplinary Plant Group, University of Missouri, Columbia, MO, United States

**Keywords:** SUMOylation, SUMO isoforms, SRFR1, basal defenses, *PstDC3000*

## Abstract

Steady-state SUMOylome of a plant is adjusted locally during developmental transitions and more globally during stress exposures. We recently reported that basal immunity in *Arabidopsis thaliana* against *Pseudomonas syringae* pv *tomato* strain DC3000 (*PstDC3000*) is associated with strong enhancements in the net SUMOylome. Transcriptional upregulations of SUMO conjugases, suppression of protease, and increased SUMO translations accounted for this enhanced SUMOylation. Antagonistic roles of SUMO1/2 and SUMO3 isoforms further fine-tuned the SUMOylome adjustments, thus impacting defense amplitudes and immune outcomes. Loss of function of *SUPPRESSOR OF rps4-RLD1* (*SRFR1*), a previously reported negative regulator of basal defenses, also caused constitutive increments in global SUMO-conjugates through similar modes. These suggest that SRFR1 plays a pivotal role in maintenance of SUMOylation homeostasis and its dynamic changes during immune elicitations. Here, we demonstrate that SRFR1 degradation kinetically precedes and likely provides the salicylic acid (SA) elevations necessary for the SUMOylome increments in basal defenses. We show that SRFR1 not only is a SUMOylation substrate but also interacts *in planta* with both SUMO1 and SUMO3. In *sum1* or *sum3* mutants, SRFR1 stabilities are reduced albeit by different modes. Whereas a *srfr1 sum1* combination is lethal, the *srfr1 sum3* plants retain developmental defects and enhanced immunity of the *srfr1* parent. Together with increasing evidence of SUMOs self-regulating biochemical efficiencies of SUMOylation-machinery, we present their impositions on SRFR1 expression that in turn counter-modulates the SUMOylome. Overall, our investigations reveal multifaceted dynamics of regulated SUMOylome changes via SRFR1 in defense-developmental balance.

## Introduction

In higher eukaryotes, pivotal roles of post-translational modifications (PTMs) balance protein expressions, activities, interacting partners, localization, and proteostasis (Walsh et al., 2005; Millar et al., 2019). With structural similarities to ubiquitin, the SMALL UBIQUITIN-LIKE MODIFIERs (SUMOs) covalently attach to lysine (K) residues of substrates through a cascade of enzymatic reactions biochemically similar to ubiquitination cycles and termed as SUMOylation (Geiss-Friedlander and Melchior, 2007; Flotho and Melchior, 2013; Zhao, 2018). The partially conserved motif, ψ–K–X–D/E (ψ = hydrophobic amino acid, X = any amino acid, and D/E = aspartate/glutamate), most often harbors the SUMOylated lysine residue. SUMOs can also facilitate non-covalent protein–protein associations requiring the essential presence of hydrophobic core-containing SUMO-interaction motifs (SIMs), K–X_3__–__5_–[V/I]–[I/L]–X_3_–[D/E/Q/N]–[D/E]_2_, in the cognate recipient (Hannich et al., 2005). Computational studies identify SUMOylation-annotated candidates as strategic central relay players of protein–protein interaction webs, and with noted predominance of DNA-modification enzymes and transcription factors (TFs), transcriptional processes are especially modulated (Duan and Walther, 2015).

Maintenance of SUMOylome homeostasis necessitates tight coordination of SUMO-conjugation/deconjugation cycles (Kurepa et al., 2003; Morrell and Sadanandom, 2019). SUMOylation requires the availability of processed SUMOs, with exposed diglycine (GG) residues at their C-terminus. SUMO proteases generate these conjugation-proficient SUMOs from precursors that contain extended C-terminus residues. The mature SUMOs through an ATP-dependent AMP–SUMO intermediate formation is linked via thiol–ester bond to the catalytic cysteine of SUMO E1 ACTIVATING ENZYME (SAE), a heterodimer of two subunits SAE1 and SAE2. SUMO E2 CONJUGATING ENZYME (SCE) then acquires the SUMO moiety from SAE via trans-esterification. Although SCE is capable of direct SUMOylation, its binding to SUMO E3 ligases, which also simultaneously interact with targeted substrates, augments the process and imparts specificity (Gareau and Lima, 2010). SUMOs are also self-SUMOylated at internal lysines forming poly-SUMO chains. These are catalyzed by SUMO E4 ligases that belong to the PIAL (protein inhibitor of activated stat-like) class of proteins (Tomanov et al., 2014). Some SUMO proteases remove SUMOs from SUMOylated substrates or disintegrate polySUMO chains, thus recycling free SUMOs (Chosed et al., 2006; Colby et al., 2006; Morrell and Sadanandom, 2019).

Insights from studies on the model plant *Arabidopsis thaliana* have provided vital clues into the complexity of SUMOylation and its genetic link to diverse cellular processes. *Arabidopsis* SUMO-machineries are mostly encoded by multiple genes showing tissue/stage-dependent, stress-inducible expressions and interaction/modification specificities with substrates. Although *A. thaliana* genome encodes eight SUMO isoforms, only four (*SUM1*, -*2*, -*3*, and -*5*) are expressed (van den Burg et al., 2010). SUMO1 and -2 proteins share considerable sequence identity (94%) and, in several instances, are functionally redundant or additive and surprisingly in some responses contrasting (Saracco et al., 2007; van den Burg et al., 2010; Castano-Miquel et al., 2011; Ingole et al., 2021b). SUMO3 and -5 proteins are more diverged with less than 50% identity to SUMO1. Simultaneous loss of *SUM1* and *SUM2* is embryonic lethal, indicating that plants require at least one functional copy of either of these isoforms. In plants, SUMO1/2 predominantly occur as free non-conjugated forms, which upon exposure to heat, peroxide, or ethanol stress are rapidly utilized for SUMOylating target proteins (Kurepa et al., 2003; van den Burg et al., 2010). The *Arabidopsis sum3* mutant is viable with mild late-flowering phenotype (van den Burg et al., 2010). SUMO3, unlike SUMO1/2, cannot form poly-SUMO chains *in vitro*, is barely detected in plant extracts, and shows little or no change to heat shock treatments (Kurepa et al., 2003; Chosed et al., 2006; Colby et al., 2006; Budhiraja et al., 2009; van den Burg et al., 2010). SUMO1/2 but not SUMO3-modified targets are efficiently deconjugated by ULPs (Chosed et al., 2006; Colby et al., 2006). Lastly, SUMO1 and SUMO3 reciprocally influence each other’s conjugation efficiencies *in vitro*, implying functional cooperativity (Ingole et al., 2021b). HIGH PLOIDY 2/METHYL METHANE SULFONATE 21 (HPY2/MMS21) and SAP and MIZ 1 (SIZ1) remain the two well-characterized SUMO E3 ligases in *Arabidopsis*. Multiple *Arabidopsis* SUMO proteases have been identified till date and include EARLY IN SHORT DAYS 4 (ESD4), its closest homologs ELS1/2 (ESD4-LIKE SUMO PROTEASE1/2), OVERLY TOLERANT TO SALT 1/2 (OTS1/2), SUMO-PROTEASES RELATED TO FERTILITY 1/2 (SPF1/2), and DE-SUMOYLATING ISOPEPTIDASES (DeSIs), among others (Kurepa et al., 2003; Murtas et al., 2003; Chosed et al., 2006; Colby et al., 2006; Kong et al., 2017; Orosa et al., 2018). However, only a fraction of these remain functionally characterized (Morrell and Sadanandom, 2019; Srivastava et al., 2021).

In broadly understood layers of plant defenses, conserved molecular signatures or pathogen-associated molecular patterns (PAMPs) present on microbes are sensed by extracellular transmembrane pattern-recognition receptors (PRRs) to transduce downstream induction of defense-associated genes (Jones and Dangl, 2006; Bentham et al., 2020). This route of immune signaling comprise the PAMP-triggered immunity (PTI). Intracellular perception of a pathogen attack is performed by strategically deployed resistance (R) proteins that directly or indirectly sense manipulations by the invader-secreted effectors. Defense responses elicited downstream of these perceptions are termed as effector-triggered immunity (ETI) and include heightened production of defensive hormone salicylic acid (SA), and prolonged and aggravated expression of PTI-responsive markers such as *PATHOGENESIS-RELATED PROTEINS* (*PRs*) and SA-biosynthesis *SALICYLIC ACID-DEFICIENT 2/ISOCHORISMATE SYNTHASE 1* (*SID2/ICS1*) gene, among others. Basal and ETI mediated by the Toll–interleukin1 (TNL)-type of R proteins require ENHANCED DISEASE SUSCEPTIBILITY 1 (EDS1) to potentiate SA-based defenses (Aarts et al., 1998; Falk et al., 1999).

Disturbances in SUMO pathway affect immune responses often with developmental costs to the host (Lee et al., 2007; van den Burg et al., 2010; Bailey et al., 2016; Orosa et al., 2018; Verma et al., 2018). Studies overall suggest that SUMO1/2 suppresses whereas SUMO3 potentiates immunity primarily through modulation of SA-signaling networks (van den Burg et al., 2010; Saleh et al., 2015; Ingole et al., 2021b). However, adjustments of a host SUMOylome in response to pathogen attack or in SUMOylation-perturbed mutants present a more complicated involvement not only of SUMO isoforms but also of SUMO-machineries. For example, globally reduced SUMOylome in *siz1-2* or enhanced SUMO1/2-conjugates in *esd4-2* or *ots1 ots2* both lead to elevated SA levels with constitutive activation of defenses (Villajuana-Bonequi et al., 2014; Bailey et al., 2016). ESD4 and SIZ1 are SUMOylated and non-covalently bind SUMOs, implying that their activities are self-regulated by SUMOylation (Miller et al., 2010; Mazur et al., 2017). Our studies recently identified both positive and negative immune regulators as differentially SUMOylated candidates upon a pathogen attack (Ingole et al., 2021a). Further, we also revealed that SUMO-conjugation efficiencies are affected by the crosstalk between SUMO isoforms independent of their covalent-modification activities. Taken together, how a plant maintains SUMOylome homeostasis, performs response-appropriate modifications on substrates, and prevents fitness costs remains uncharacterized.

We recently showed that in the autoimmune mutant *srfr1-4* constitutive and during *PstDC3000* infections on wild-type plants progressive, increments of SUMO1/2 conjugations were observed, respectively (Ingole et al., 2021a). This implies a genetic link of SRFR1 to SUMOylome maintenance and responsive adjustments. Here, we reveal that SRFR1 potentiates *PstDC3000*-induced SUMOylome changes, undergoing transient instability that kinetically precedes SA accumulations and global SUMOylome enhancements. With known link in suppressing *SID2/ICS1* expressions, SRFR1 reduction thus likely provides the SA stimulus previously shown to enhance SUMO-conjugations (Bailey et al., 2016). We deduce here that in addition to being a SUMOylation candidate, SUMOylome perturbation also reciprocally affects SRFR1 expressions at both transcriptional and post-transcriptional levels. Further, enhanced basal defenses or SUMO1/2-conjugation increments are negligibly *SUM3*-dependent in *srfr1-4* plants. Overall, our investigations here present SRFR1 role in SUMOylome homeostasis and perturbations providing fine-tuning of immune amplitudes.

## Materials and Methods

### Plant Materials and Growth Conditions

Mutants of *A. thaliana* used here, namely, *sum1-1*, *sum3-1*, *sum1-1 sum3-1*, *srfr1-4*, *sid2-1*, *srfr1-4 eds1-2*, *srfr1-4 snc1-11*, and *esd4-2*, have been described earlier (van den Burg et al., 2010; Bhattacharjee et al., 2011; Villajuana-Bonequi et al., 2014; Ingole et al., 2021b). Plants were propagated under short day (SD; 8-h light; 16-h dark) conditions at 22°C (or 24°C for *PstDC3000* infection-based assays) with 70% relative humidity in controlled growth chambers with a light intensity of 100 μmol photons m^–2^ s^–1^. To generate double mutants *srfr1-4 sid2-1*, *srfr1-4 sum3-1*, or *HA-SRFR1* expressing plants in SUMOylation-disturbed (*sum1-1*, *sum3-1*, *esd4-2*, or *esd4-2 sum3-1*) mutants, indicated plants discussed in the respective results sections were crossed. From the segregating F2 or F3 populations, the desired genotype was identified and propagated for experiments. To generate plants co-expressing *EDS1-YFP* and *His-H89R-SUM1*, the parental *EDS1-YFP* (Garcia et al., 2010) and *His-H89R-SUM1* (Miller et al., 2010) plants were crossed. F1 plants were verified for the presence of both transgenes before analysis. Primers for genotyping are listed in Supplementary Table 1.

### Salicylic Acid Measurements

Free and total SA (SA + glucose-conjugated, SAG) measurements were performed according to Defraia et al. (2008) using the *Acinetobacter* sp. ADPWH_*lux* biosensor system. Briefly, 100 mg of frozen tissue was homogenized in 250 μl of acetate buffer (0.1 M, pH 5.6) and clarified by centrifugation at 12,000 rpm for 15 min to remove cell debris. A 100 μl of the supernatant was kept for measuring free SA, while a similar volume was treated with 6 U of β-glucosidase (Sigma-Aldrich, St. Louis, MO, United States) for 90 min at 37°C for total SA determination. A 20 μl aliquot of plant extract was mixed with 50 μl of *Acinetobacter* suspension (grown to OD_600_ = 0.4) along with 60 μl of fresh Luria–Bertani (LB) broth. Standard curve was generated with *sid2-1* tissue extracts spiked with known amounts of SA (Sigma-Aldrich, United States). After incubation for 1 h at 37°C, luminescence was measured using a Luminometer (POLARStar Omega, BMG Labtech, Ortenberg, Germany). At least three biological replicates were used for each measurement, and data were reported as mean ± SD.

### Total RNA Extraction and Quantitative Real-Time PCR

Total RNA extraction from plant tissues was performed using RNAiso Plus reagent (Takara, Maebashi, Japan), DNase-treated (TURBO^TM^ DNA-free kit, Thermo Fisher Scientific, Wilmington, DE, United States), and then reverse transcribed (iScript^TM^ cDNA Synthesis Kit, Bio-Rad, Hercules, CA, United States) according to the manufacturer’s instructions. All qPCR primers used here are listed in Supplementary Table 1. Real-time PCRs were performed with HOT FIREPol EvaGreen qPCR Mix Plus (ROX) (Solis BioDyne, Tartu, Estonia) as suggested by the manufacturer and on a QuantStudio 6 Flex machine (Applied Biosystems, Foster City, CA, United States). Expressions were normalized to the endogenous control *MON1* (*At2g28390*) levels and calculated according to the (PCR efficiency)^–ΔΔ^ ^Ct^ formula (Kim et al., 2010). Each experiment was repeated at least twice with three biological and technical replicates.

### Protein Extraction, Immunoprecipitation, and Immunoblotting

Sample processing for anti-SUMO1/2 immunoblots is described in Ingole et al. (2021a). In brief, tissues snap frozen and stored in –80°C were homogenized in protein extraction buffer (PEB) [50 mM of Tris–HCl pH 8.0, 8 M of urea, 50 mM of NaCl, 1% w/v NP-40, 0.5% w/v sodium deoxycholate, 0.1% w/v sodium dodecyl sulfate (SDS) and 1 mM of EDTA, and 20 mM of *N*-ethylmaleimide (NEM)] containing freshly added 1 × plant protease inhibitors (Sigma-Aldrich, United States) and 2% w/v polyvinylpolypyrrolidone (PVPP). For anti-HA, anti-EDS1, anti-GFP, or anti-PR1/2 immunoblots, tissues were ground with 6 M of urea. Clarified homogenates of extracts were mixed with loading dye, boiled, resolved in SDS–polyacrylamide gel electrophoresis (SDS-PAGE), and transferred to polyvinylidene difluoride (PVDF) membrane via wet transfer. After blocking with 5% w/v non-fat skimmed milk powder in 1 × Tris-buffered saline (TBST; containing 0.1% w/v Tween^®^ 20), membrane was incubated overnight with indicated primary antibodies [anti-SUMO1 (Abcam, Cambridge MA, United States), anti-PR1/2 (Agrisera, Vännäs, Sweden), anti-EDS1 (custom generated against full-length *Arabidopsis* EDS1 from BioBharati LifeScience, Kolkata, India), anti-HA (Sigma, United States), or anti-GFP (BioBharati LifeScience, India)] in 1 × TBST. Membranes were washed thrice the next day with TBST, incubated at room temperature (RT) with secondary antibodies conjugated with horseradish peroxidase (HRP) (Santa Cruz Biotech, Dallas, TX, United States), and developed with ECL Prime western blotting kit (GE Healthcare, Chicago, IL, United States). Images were acquired with ImageQuant LAS 4000 (GE Healthcare, United States). The membranes were stained with Ponceau S and imaged to demonstrate comparable loading.

SUMO1/2-conjugate enrichments from plants expressing *EDS1-YFP* with or without *His-H89R-SUM1* were performed as earlier (Ingole et al., 2021a).

For immunoprecipitation assays between HA-SRFR1 and Myc-EDS1 or HA-PAD4 and Myc-EDS1, agro-infiltrated *Nicotiana benthamiana* leaves were used 48 h post-infiltration. Tissues were ground in chilled radioimmunoprecipitation assay (RIPA) buffer [5 mM of Tris–HCl pH 7.5, 150 mM of NaCl, 10 mM of MgCl_2_, 1 mM of EDTA, 1% NP-40, 1 mM of sodium deoxycholate, and 1 × protease inhibitor cocktail (Sigma-Aldrich, United States)] and clarified by centrifugation. Supernatant was precleared with IgG agarose beads (Sigma-Aldrich, United States) rotated at 4°C for 1 h. The mix was then centrifuged to remove non-specific proteins bound on the IgG agarose beads, and supernatant was added to anti-HA-conjugated beads (Sigma, United States). After tumbling 3 h at 4°C, the suspension was centrifuged, and agarose-bead pellet washed three times with RIPA buffer. Beads were then resuspended in loading dye and used for indicated immunoblots.

For MG132 treatments, the proteasome inhibitor at a dose of 100 μM in 10 mM of MgCl_2_, 10 mM of 2-(*N*-morpholino)ethanesulfonic acid (MES) was infiltrated into plant tissues 12 h before harvesting.

Densitometric quantifications of protein bands in immunoblots were performed with the ImageJ software.

### Construction of Clones for *Escherichia coli* SUMOylation Assay

*SUM1* cDNA sequences in *p*CDFDuet TM1 vector were mutagenized via overlapping oligos at position 91 to incorporate arginine (R) replacing the threonine (T) residue. This resulted in SUMO1 T91R, which imparts smaller SUMOylation footprint of RGG on the modified lysine. For generating FLAG-SRFR1, cDNA sequence was directionally cloned as a *Not*I restriction fragment at the identical site of *p*FLAG-TEV vector (BioBharati LifeScience, India). For generating T7-EDS1, the cDNA sequence was cloned as a *Sal*I–*Xho*I fragment into the *p*ET28a(+) vector (Novagen, Madison, WI, United States). Details of primers used here are listed in Supplementary Table 1.

The SUMOylation assay was performed according to Okada et al. (2009). Briefly, BL21 (DE3) cells containing *p*CDFDuet TM1-SUMO1/2/3 GG form together with SCE1, or *p*CDFDuet TM1-SUMO1/2/3 AA form co-cloned with SCE1, and *p*ACYCDuet TM1-SAE2 and SAE1 were co-transformed with either *p*FLAG-SRFR1 or *p*T7-EDS1 plasmid. Heterologous protein expression was induced with 0.5 mM of IPTG overnight at 28°C. The cell pellet harvested the next day was resuspended in phosphate-buffered saline (0.137 M of NaCl, 0.0027 M of KCl, 0.01 M of Na_2_HPO_4_, and 0.0018 M of KH_2_PO_4_, pH 7.4), lysed by boiling in 1 × loading dye, and then used for immunoblots with anti-FLAG (Sigma, United States) or anti-T7 antibodies (Merck Millipore, Kenilworth, NJ, United States).

### Sample Preparation and Liquid Chromatography–Tandem MS Analysis for Determination of SUMOylation Sites

Processing for in-gel trypsin digestion and MS analysis was according to Ingole et al. (2021a). Briefly, proteins from *in vitro* SUMOylation reaction were separated on 6% SDS-PAGE, stained with Coomassie brilliant blue (CBB R-250) solution, and destained; and gel slices were excised as 1-mm^3^ pieces with sterile surgical blade. After processing as earlier, trypsin digestion (Promega, Madison, WI, United States) was performed; samples were desalted with C18 tips (Thermo Fisher Scientific, United States) according to manufacturer’s instructions and dried in a speed-vac centrifuge. Tandem MS (MS/MS) analysis was done using TripleTOF^®^ 5600+ (AB SCIEX, Redwood City, CA, United States) mass spectrometer instrument. Raw MS data files were searched for peptide sequences (MASCOT software, Matrix Science, Boston, MA, United States) against the *A. thaliana* protein database^1^.

### Construction of Bimolecular Fluorescence Complementation, GFP-Tagged SUM1 GG/AA Vectors, and *in planta* Assays

Bimolecular fluorescence complementation (BiFC) vectors for SRFR1, EDS1, SUMO1 GG/AA, and SUMO3 GG/AA have been described earlier (Bhattacharjee et al., 2011; Ingole et al., 2021b). Clones were introduced into *Agrobacterium tumefaciens* GV3101 strain via electroporation. For *in planta* interaction assays, *Agrobacterium* strains harboring the indicated BiFC vectors were cultured overnight in LB broth, centrifuged, and resuspended in 10 mM of MgCl_2_, 10 mM of MES containing 150 mM of acetosyringone. After induction for 3–4 h, indicated combinations were mixed at equal bacterial density and infiltrated in fully expanded leaves of 4-week-old *N. benthamiana*. At 48 h post-infection (hpi), tissue sections from the infiltrated area were excised and imaged under a 40 × oil objective in SP8 Leica confocal microscope (Leica microsystems, Wetzlar, Germany) using the fluorescein isothiocyanate (FITC) filter (488-nm argon laser).

### Construction of HA-SRFR1^K325R^, HA-SRFR1^K427R^, HA-SRFR1^K325R + K427R^, GFP-SUMO1 GG/AA Forms, or SUMO3, Myc-EDS1^WT^, or Myc-EDS1^K478R^ Expression Vectors and Generation of Transgenic Plants

The construction of HA-SRFR1 expressing native promoter-driven HA-epitope tagged genomic clone of *SRFR1* has been described earlier (Kim et al., 2010). With the use of this vector as a backbone, the indicated lysine residues were converted to arginine using overlapping primers (listed in Supplementary Table 1). *p*DONR201 clone of SUMO1 GG or AA, SUMO3-GG described earlier (Ingole et al., 2021b), was subcloned via Gateway reaction into *p*SITE-2CA binary vector that carries an N-terminal GFP tag (Chakrabarty et al., 2007). The above clones were confirmed by sequencing and then electroporated into *Agrobacterium* GV3101 strain. Transformants obtained were cultured; and *srfr1-4* or *sum3-1*, as appropriate, was transformed via floral-dip method (Clough and Bent, 1998). Transgenic plants were selected on kanamycin (for HA-SRFR1 or GFP-SUMO3 transformations) containing plant growth media and then propagated to T2 and T3 generations and genotyped. Simultaneously, expression of GFP-SUMO3 was also determined via anti-GFP immunoblots. *GFP-SUM3* Line#1 was crossed to *HA-SRFR1* expressing plants and F2/F3 population genotyped for *sum3-1*, *srfr1-4*, mutations, and *HA-SRFR1* or *GFP-SUM3* transgene homozygosity. The plants were then used for further analysis, as indicated.

For co-expression assays with *HA-SRFR1*, *Agrobacterium* strains with GFP alone (*p*SITE-2CA) or GFP-*SUM1* GG or AA forms (in *p*SITE-2CA backbone) was co-infiltrated as above. Total protein from infiltrated tissues was isolated at 48 hpi and used for immunoblot with anti-HA antibodies.

Construction of Myc-EDS1^WT^ binary has been described earlier (Bhattacharjee et al., 2011). With the use of the *p*DONR201-EDS1^WT^, the lysine 478 residue was mutated to arginine (EDS1^K478R^) by overlap PCR. This was then subcloned into *p*BA-Myc binary vector via Gateway-based cloning. Plant transformations with *eds1-2* were done as earlier. Independent transgenic lines were selected on 1/2 MS + Basta (10 μg/ml) plates and grown to F3 for assays. Expression of recombinant protein was checked with anti-Myc immunoblots. Primers for various cloning are listed in Supplementary Table 1.

### *In planta* Qualitative and Quantitative Bacterial Growth Assay

Bacterial growth assays were performed according to standardized protocol in Kim et al. (2010). Briefly, fully expanded leaves of 3- to 4-week-old SD-grown plants were infiltrated with a needleless syringe at a bacterial density of 10^6^ cfu/ml (half-leaf infiltrated for qualitative disease symptom assay) or 5 × 10^4^ cfu/ml (full leaf infiltrated for quantitative bacterial growth assays) with virulent *PstDC3000* or avirulent *PstDC3000* (*avrRps4*) strains. For disease symptom determinations, infiltrated leaves were detached 4 days post-infiltration (dpi) and imaged. For quantitative measurements on bacterial growth, leaf disks of defined area were harvested at 0 and 3 dpi, macerated in 10 mM of MgCl_2_, and plated with serial dilution on appropriate antibiotic containing media plates. Anti-SUMO1/2 immunoblot on Col-0, *sum1-1*, *sum2-1*, and *sum3-1* post-*PstDC3000* challenge (10^6^ cfu/ml bacterial inoculum) was performed at 24 hpi.

## Results

### *SRFR1* Instability Precedes Global SUMO1/2ylation and Salicylic Acid Elevations During Basal Defenses

**FIGURE 1:**
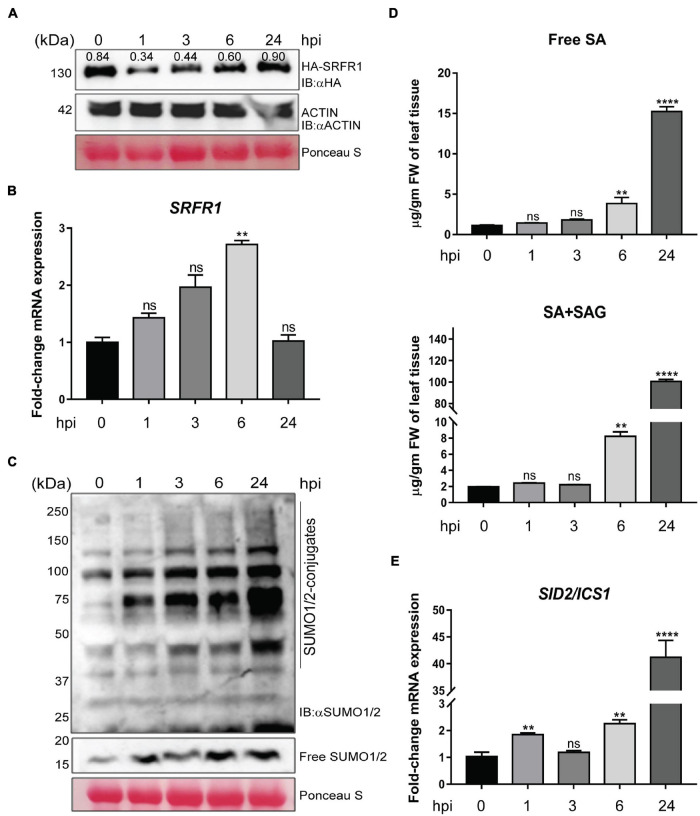
SRFR1 protein instability promotes salicylic acid (SA) and SUMOylome elevations in basal defenses. At progressive time points (hpi, hours post-infection) upon *PstDC3000* challenge **(A)** immunoblot of HA-SRFR1, **(B)** expression levels of *SRFR1* transcripts, **(C)** SUMO1/2-conjugates and free SUMO1/2 levels, **(D)** free SA and total SA + SAG levels, and **(E)** expression of *SID2/ICS1*. Blots were probed with anti-HA or anti-SUMO1/2 antibodies, as indicated. Ponceau S-stained membrane or anti-actin immunoblot shows comparable protein loading across samples. Numbers above the HA-SRFR1 protein bands in panel **(A)** are densitometric quantification values relative to actin expression levels in the same extracts. Transcript abundance is relative to internal control *MON1* gene expressions and presented as fold-change relative to uninfected (0 hpi) samples (*n* = 3). Statistical significance is by pairwise comparison with 0-hpi sample with Student’s *t*-test (**p < 0.01, ****p < 0.0001, and ns = not significant).

Recently, we demonstrated that SRFR1 regulates *Arabidopsis* SUMOylome maintenance (Ingole et al., 2021a). The *srfr1-4* plants contained elevated levels of SUMO1/2-conjugates and displayed upregulated SUMO conjugation-promoting enzymes (*SCE1*, *SAE1/2*, *SIZ1*, and *HPY2*) and downregulated SUMO-protease (*ESD4* and *ELS1*) expressions with preferential recruitment of *SUM1* transcripts on polysomes. Though SUMOylome increments were also observed when wild-type (Col-0) plants were challenged with *PstDC3000*, SRFR1 functions were not directly attributed to these responses. To determine this, *HA-SRFR1/srfr1-4* plants (referred hereafter as *HA-SRFR1*) expressing HA-epitope tagged SRFR1 protein from its native promoter were used. These plants were shown earlier to complement the growth and defense defects of *srfr1-4* (Kim et al., 2010). *HA-SRFR1* plants were challenged with *PstDC3000*, and tissue extracts from progressive time points were immuno-probed with anti-HA antibodies. Decrease in SRFR1 protein levels was noted as early at 1 hpi and persisted moderately at 3 and 6 hpi, after which gradual restoration to near wild-type levels was noted by 24 hpi (Figure 1A). Transcripts of *SRFR1* showed progressive upregulation increasing from 1 to 6 hpi (∼2- to 2.5-fold) and restored to endogenous levels by 24 hpi (Figure 1B). Immunoblot with anti-SUMO1/2 antibodies showed gradual increments in SUMO1/2-conjugations from 1 to 24 hpi (Figure 1C). Bailey et al. (2016) demonstrated that SA application enhances SUMO1/2-conjugations in Col-0. With previously known functions in suppressing SA-based defenses, to determine whether transient instability of SRFR1 preceded SA increase to cause SUMOylome changes, we measured free SA and its glucose-conjugate (SAG) levels in the above extracts. Significant increase in free and total (SA + SAG) levels (∼4- and ∼8-fold, respectively) was first noted at 6 hpi, and by 24 hpi, drastic elevations were observed (∼15- and ∼55-fold, respectively) (Figure 1D). This moderately correlated to the upregulation in *SID2/ICS1* expressions (Figure 1E). Taken together, these data suggest that transient decrease in SRFR1 protein is linked to SUMOylome perturbation via SA-signaling routes.

**FIGURE 2:**
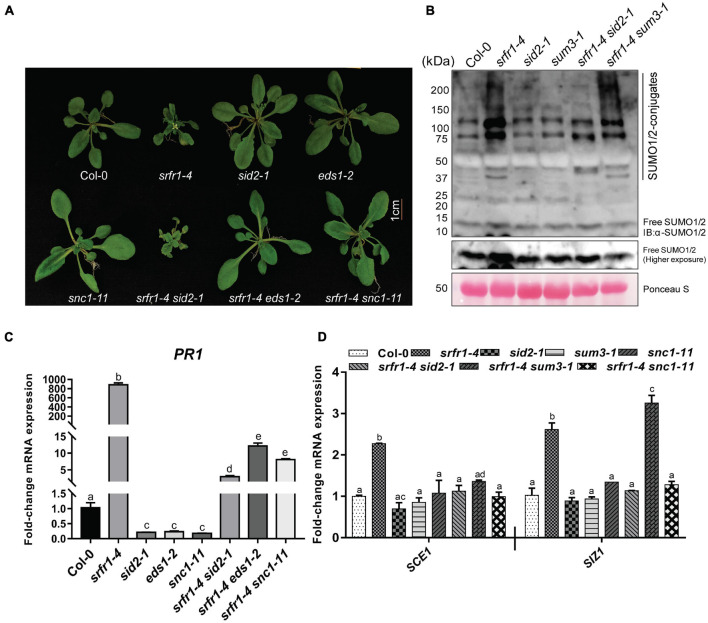
SUMOylome enhancements but not growth defects in *srfr1-4* are *SID2/ICS1*-dependent. **(A)** Growth phenotypes of 4-week-old Col-0, *srfr1-4*, *srfr1-4 sid2-1*, *srfr1-4 eds1-2*, *srfr1-4 snc1-11*, *sid2-1*, *eds1-2*, and *snc1-11* plants. Scale bar = 1 cm. **(B)** Immunoblot of SUMO1/2-conjugates and free SUMO1/2 in indicated plant genotypes. Protein extracts were probed with anti-SUMO1/2 antibodies. Ponceau S staining is indicative of loading controls. Transcript abundance of **(C)** *PR1*, **(D)** *SCE1*, or *SIZ1*, is relative to control *MON1* expression in the indicated genotypes (*n* = 3). Values are mean ± SD and shown as fold-change relative to Col-0 levels. Different letters indicate statistical differences according to *post hoc* Tukey’s test (*p* < 0.05).

To directly correlate SA increase due to *SID2/ICS1* upregulation as the causal factor of SUMOylome enhancements in *srfr1-4*, we generated *srfr1-4 sid2-1* plants by genetic crossing. Unlike *srfr1-4 eds1-2* that are developmentally similar to Col-0, *srfr1-4 sid2-1* plants retained stunted stature-like *srfr1-4* (Figure 2A). Thus, growth deficiencies in *srfr1-4* are *SID2/ICS1*-independent. Total (SA + SAG) and free SA levels in *srfr1-4 sid2-1* resembled those of Col-0 (Supplementary Figure 1A). To determine global SUMOylome profile in *srfr1-4 sid2-1*, anti-SUMO1/2 immunoblots were performed. We also included *srfr1-4 snc1-11* plants in this analysis. The *snc1-11* is a knockout mutation in *SNC1*, the *R* gene responsible for growth abnormalities in *srfr1-4* (Kim et al., 2010). The *srfr1-4 snc1-11* plants developmentally resemble Col-0 but retain intermediate upregulation of defense-associated markers and enhanced resistance than *srfr1-4* (Kim et al., 2010). We noted that *sid2-1* or *snc1-11* mutation completely abolished SUMO1/2-conjugate or free SUMO1/2 enhancements of *srfr1-4* to Col-0 levels (Figure 2B and Supplementary Figure 1B). Further, elevated free SA and total SA were also restored to Col-0 levels in the *srfr1-4 eds1-2* or *srfr1-4 snc-11* plants (Supplementary Figure 1A). Additionally, increased expressions of SUMOylation-promoting genes shown earlier (*SIZ1*, *SCE1*, or *SUM3*) and *PR1* transcripts were considerably downregulated to Col-0 levels in *srfr1-4 sid2-1* plants (Ingole et al., 2021a; Figures 2C,D and Supplementary Figure 1C). Expression of *SUM1* remained unaltered in all plant genotypes. These results implied that SA upregulation via heightened expression of *SID2/ICS1* was mainly responsible for SUMOylome enhancements in *srfr1-4* in *SNC1*- and *EDS1*-dependent manner.

### *SRFR1* Is a SUMOylation Candidate

**FIGURE 3:**
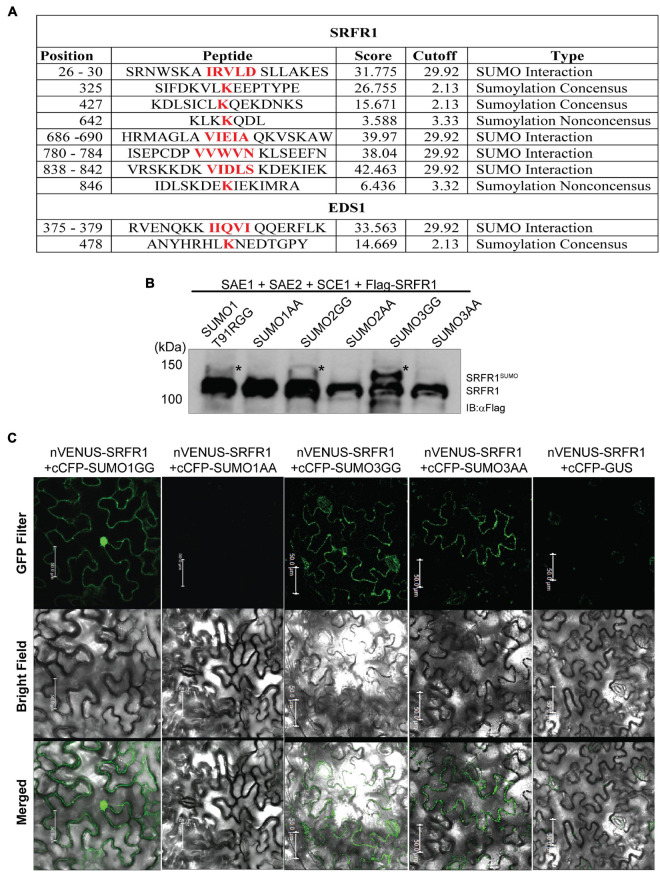
SRFR1 is a SUMOylation and SUMO-binding candidate. **(A)** Prediction of putative SUMOylation and SIM motifs in SRFR1 and EDS1. *In silico* prediction was performed using SUMOsp2.0 tool. **(B)** SRFR1 is SUMOylated by SUMO1 T91R, SUMO2, or SUMO3 GG forms in *Escherichia coli* SUMOylation reconstitution system. Immunoblot was probed with anti-FLAG antibodies to detect FLAG-SRFR1. Migration position of molecular weight standards (in kDa), SUMOylated or non-SUMOylated SRFR1 (SRFR1^SUMO^ or SRFR1, respectively), is indicated. **(C)** Bimolecular fluorescence complementation (BiFC) panels of co-expressed nVenus-SRFR1 with cCFP-clones of SUMO1 GG/AA, SUMO3 GG/AA, or negative control GUS (β-glucuronidase). Images were acquired using a confocal microscope with GFP or bright-field filters. Merged images of YFP with bright field are shown. Scale bar = 50 μm.

*SRFR1* harbors several intrinsically disordered regions (IDRs) especially between its multiple TPRs and the C-terminal domain (Gassmann and Bhattacharjee, 2012). Protein with IDR features often has various interacting partners adjusted according to the cellular signaling needs (Haynes et al., 2006; Liu et al., 2009). Most often, IDRs are also enriched for motifs that attract PTM changes (Gao and Xu, 2012; Narasumani and Harrison, 2018). In SRFR1, two sites that match canonical SUMOylation motifs (LK^325^EE and LK^427^QE) are predicted by the SUMOsp2.0 tool (Ren et al., 2009; Figure 3A). Plus, at least two each of high-scoring SIMs and non-consensus SUMOylation sites are also present in SRFR1. To test whether SRFR1 is SUMOylated by the *Arabidopsis* SUMO isoforms, we utilized the *Escherichia coli* SUMOylation reconstitution system, with minor modifications (Okada et al., 2009). The N-terminal 91st residue N-terminal to GG in SUMO1 was modified from threonine to arginine (T91R) to facilitate smaller SUMO footprints on targets and improved identification via MS. SUMO3 contains a relatively proximal arginine at the 88th position to the diglycine G^92^G^93^ ends. MYB30, a previously known target tested by Okada et al. (2009), was SUMOylated by SUMO1 T91R variant (Supplementary Figure 2). SRFR1 cDNA sequences were co-expressed with a FLAG-epitope tag (FLAG-SRFR1) in the presence of SUMO1 T91R, SUMO2, or SUMO3 SUMOylation-competent (containing diglycine, GG) or SUMOylation-deficient (diglycine replaced by dialanine, AA) isoforms. Induced *E. coli* extracts probed with anti-FLAG antibodies showed a slower migrating band than FLAG-SRFR1 in the presence of only GG, but not AA forms of the co-expressed SUMOs (Figure 3B). This band was more intense when SUMO3 was co-expressed than with SUMO1/2. These results suggested that SRFR1 is a potential SUMOylation candidate of multiple SUMO isoforms.

We subjected the slower migrating SRFR1 protein bands to trypsin digests and liquid chromatography (LC)–MS/MS analysis. SUMO1, but not SUMO2 or SUMO3, footprints on selective lysine residues of SRFR1 were detected in the identified peptides (Supplementary Figure 3). Remarkably, K^325^ in the predicted LK^325^EE SUMOylation motif was identified with SUMO1-modifications. In addition, SRFR1 K^229^ was also covalently modified by SUMO1, though conserved features of a SUMOylation motif were absent for this lysine (CK^229^PC). These results demonstrated SRFR1 as a SUMOylation candidate, at least in the *E. coli* system. To test the functional relevance of *in silico* predicted SRFR1 SUMOylation sites, we generated native promoter-driven HA-SRFR1 expressing transgenic plants containing K325R, K427R, or K325R + K427R mutations (named as HA-SRFR1^K325R^, HA-SRFR1^K427R^, or HA-SRFR1^K325R + K427R^, accordingly), in the *srfr1-4* background. Functional complementation of the expressed SRFR1 variant is expected to abolish growth defects of *srfr1-4*. Transgenic plants expressing HA-SRFR1^K325R^, HA-SRFR1^K427R^, or HA-SRFR1^K325R + K427R^ versions of SRFR1 restored wild-type growth in *srfr1-4* (Supplementary Figure 4). These results implied that R substitutions of at least these K residues do not affect SRFR1 function. With one additional lysine residue (K^229^) identified in SRFR1 as a potential SUMO-acceptor, similar mutational and complementation studies are needed to decipher its functional relevance. Detection *in planta* of SUMO1-modifications on K^721^ of TPR1, which is not a predicted SUMOylation site, encourages possibility of a non-conventional motif in SUMO-influences on SRFR1 (Niu et al., 2019).

To evaluate non-covalent binding of SUMOs to SRFR1, we utilized the BiFC assays. *N. benthamiana* leaves transiently expressing SRFR1 showed restoration of YFP signal with SUMO1 or SUMO3 co-expressed as GG (SUMOylation-competent) forms (Figure 3C). When SUMOs were expressed as AA (SUMOylation-deficient) forms, only SUMO3 but not SUMO1 bound SRFR1. As controls, neither SRFR1 nor SUMOs interacted with the negative control beta-glucuronidase (GUS) protein or the corresponding empty vector (Figure 3C and Supplementary Figure 5). These results suggest that SRFR1 is a likely SUMOylation target as well as SUMO-binding protein.

### EDS1 K^478^, a Predicted SUMOylated Residue, Is Essential for Interaction With *SRFR1*

**FIGURE 4:**
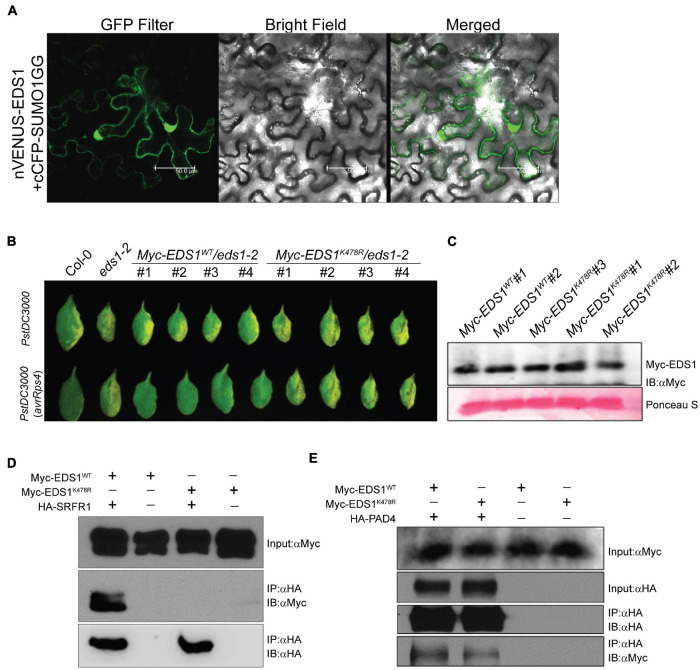
EDS1^K478R^ does not interact with SRFR1 and is functionally deficient in supporting defenses. **(A)** EDS1 interaction with SUMO1 in bimolecular fluorescence complementation (BiFC) assays. YFP reconstitution was detected with a confocal microscope equipped with a GFP filter. Also shown are merge of GFP filter and bright-field images. Scale bar = 50 μm. **(B)** Disease symptoms on independent transgenic plants expressing Myc-EDS1^WT^ or Myc-EDS1^K478R^ in *eds1-2* background infected with virulent *PstDC3000* (*top panel*) or avirulent *PstDC300* (*avrRps4*) (*bottom panel*) strains. **(C)** Expression levels of Myc-EDS1^WT^ and Myc-EDS1^K478R^ in independent transgenic lines. Total protein extracts were immunoblotted with anti-Myc antibodies. **(D)** Immuno-enrichment of HA-SRFR1 co-elutes Myc-EDS1^WT^ but not Myc-EDS1^K478R^. Co-expression was performed in *Nicotiana benthamiana* leaves, and anti-HA immuno-enrichments were probed with anti-HA or anti-Myc antibodies, as indicated. **(E)** Both Myc-EDS1^WT^ and Myc-EDS1^K478R^ interact similarly with HA-PAD4. PAD4 was pulled down with anti-HA antibodies and probed for Myc-EDS1 or Myc-EDS1^K478R^ presence.

To test for possible functional relevance of SRFR1 SIMs, we utilized its previously known interaction with EDS1 (Bhattacharjee et al., 2011). As a negative immune regulator, SRFR1 likely sequesters EDS1 from activation of defenses. A high-scoring LK^478^NE matching the consensus SUMOylation motif is identified in the EDS1 protein sequence (Figure 3A). EDS1 K^478^ is located in the EP domain and is bracketed by residues R^475^ and D^481^ that form salt bridges with the corresponding loop residues in its partner SAG101 (Wagner et al., 2013). When tested in the *E. coli*–SUMOylation system, EDS1 was not SUMOylated by any of the SUMO isoforms (Supplementary Figure 6). Interestingly, in BiFC assays, co-expression of EDS1 and SUMO1 GG but not SUMO1 AA or SUMO3 GG/AA allowed reconstitution of YFP fluorescence, hinting that SUMO1 is a direct modifier of EDS1 (Figure 4A). To test the relevance of EDS1 K^478^ in plant defenses, we generated binary vectors that express CaMV 35S promoter-driven Myc-epitope tagged wild-type (Myc-EDS1^WT^) or K478R (lysine replace by arginine; Myc-EDS1^K478R^) versions of EDS1 cDNA. The binary vectors were then used to generate transgenic plants in the *EDS1*-null (*eds1-2*) background (Cui et al., 2017). Transgenic plants obtained were then tested in qualitative disease assays with the virulent *PstDC3000* or avirulent *PstDC3000* (*avrRps4*) strains. While Myc-EDS1^WT^ expression in *eds1-2* reinstated the basal defenses causing considerably lower chlorotic symptoms, Myc-EDS1^K478R^ remained similar to hyper-susceptible and collapsed *eds1-2* leaves indicative of enhanced bacterial accumulations (Figure 4B). To the avirulent *PstDC3000* (*avrRps4*) challenges, *Myc-EDS1^K478R^/eds1-2* remained as hyper-symptomatic as *eds1-2*, while *Myc-EDS1^WT^/eds1-2* displayed Col-0-like resistance. Comparable Myc-EDS1^WT^ and Myc-EDS1^K478R^ protein expressions were noted between the transgenic plants (Figure 4C). Overall, these results indicated that EDS1^K478R^ is functionally deficient in supporting defenses. To test whether interactions with SRFR1 are affected for EDS1^K478R^, we co-expressed Myc-EDS1 (wild type or K478R) with HA-SRFR1 in *N. benthamiana* leaves. Immuno-enrichment of HA-SRFR1 detected Myc-EDS1^WT^ but not Myc-EDS1^K478R^, indicating that EDS1 K^478^ is important for SRFR1 interaction (Figure 4D). Interestingly, PAD4, a known partner of EDS1 (Feys et al., 2001), when transiently co-expressed (as HA-PAD4) in *N. benthamiana* leaves showed comparable interaction with either Myc-EDS1^WT^ or Myc-EDS1^K478R^, implying that EDS1 K^478^ is not essential for their association (Figure 4E).

To detect for SUMOylated EDS1 *in planta*, we obtained F1 plants that co-expressed *EDS1-YFP* and *His-H89R-SUM1*. The crossed parents expressed *EDS1-YFP* or *His-H89R-SUM1* that functionally complement the loss of respective endogenous *EDS1* or *SUM1* (Garcia et al., 2010; Miller et al., 2010). Expression of *His_6_-SUM1-H89R* facilitates improved affinity-based enrichment of SUMO1-SUMOylated proteins. *EDS1-YFP* (control) or *EDS1-YFP*/*His-H89R-SUM1* plants were sprayed with SA to mimic defense responses and then enriched for His-SUMO1-conjugated proteins with Ni^2+^-affinity chromatography, as earlier (Ingole et al., 2021a). Eluates showed enrichment of SUMO1-conjugates and when probed with anti-GFP antibodies identified a protein band at expected migration positions for SUMOylated EDS1-YFP (EDS1^SUMO1^) only in *EDS1-YFP*/*His_6_-SUM1-H89R* but not *EDS1-YFP* samples (Supplementary Figure 7). Input extracts from both samples displayed similar levels of EDS1-YFP expression. Because of their low amounts, we were unable to perform MS analysis on the hypothesized EDS1^SUMO1^ protein bands. In a parallel approach, we transiently co-expressed Myc-EDS1^WT^ or Myc-EDS1^K478R^ with GFP-SUMO1 or His-StrepII-SUM3g (Ingole et al., 2021b) in *N. benthamiana* leaves and probed whether SUMO1/3 co-expression caused reduced migration of EDS1 indicative of its SUMOylation. Immunoblot, however, showed similar migration of Myc-EDS1^WT^ across all SUMO combinations (Supplementary Figure 8). Thus, despite our attempts, reasonable doubt whether *in planta* EDS1 is SUMOylated (on K^478^) persists, and further investigations to elucidate this are therefore warranted.

### *sum1-1* or *sum3-1* Affect Endogenous Levels and Dynamics of *SRFR1* During Basal Defenses

**FIGURE 5:**
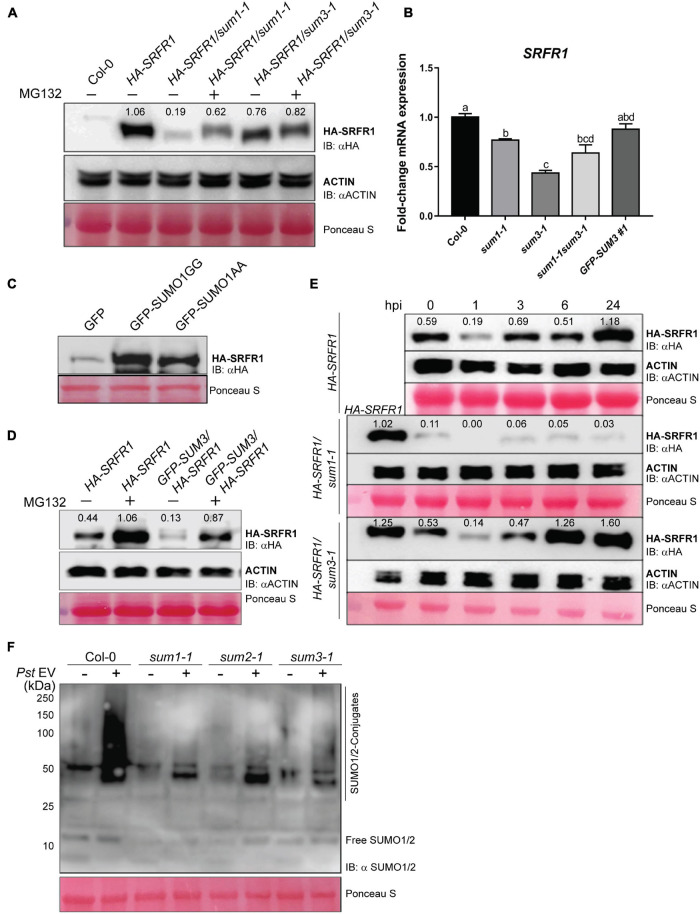
Steady-state levels of SRFR1 are lower in *sum1-1* or *sum3-1* plants. **(A)** HA-SRFR1 protein detection in Col-0 (negative control), wild-type (*HA-SRFR1*), *HA-SRFR1/sum1-1*, and *HA-SRFR1/sum3-1* plants. **(B)** Relative abundance of *SRFR1* transcripts in Col-0, *sum1-1*, *sum3-1*, *sum1-1 sum3-1*, or *GFP-SUM3* overexpressing plants. *MON1* expressions were used as the internal control, and values are mean ± SD (*n* = 3). Statistical significance is according to *post hoc* Tukey’s test (*p* < 0.05) and represented by different alphabets. HA-SRFR1 protein levels with overexpressed **(C)** GFP alone, or GFP-SUMO1 GG or AA forms. **(D)** GFP-SUMO3. **(E)** Changes in HA-SRFR1 protein at 0, 1, 3, 6, and 24 hpi *PstDC3000* (*Pst* EV) infection (hpi) in wild-type (*HA-SRFR1*), *HA-SRFR1/sum1-1*, or *HA-SRFR1/sum3-1* plants. **(F)** Accumulation of SUMO1/2-conjugates in Col-0, *sum1-1*, *sum2-1*, or *sum3-1* at 24 hpi. Immunoblots were probed with anti-HA or anti-SUMO1 antibodies as mentioned. Ponceau S staining show protein loadings. Migration position of molecular weight standards (in kDa) is indicated. MG132 treatments, where indicated, was performed at 12 h prior to respective analysis. Numbers above the HA-SRFR1 bands are densitometric quantification values relative to actin expression levels in the same extracts.

With indications of SRFR1 as a potential SUMOylation and SIM-harboring candidate, we tested whether enhanced or compromised defenses, respectively, in *sum1-1* or *sum3-1* (Ingole et al., 2021b), are the result of changes in SRFR1 levels. Toward this, we generated *HA-SRFR1/sum1-1* plants by crossing *sum1-1* to the *HA-SRFR1* line. Plants with homozygous *sum1-1* mutation displayed reduced HA-SRFR1 protein in comparison with the *HA-SRFR1* parent (Figure 5A). To determine whether the lower protein levels were due to transcriptional or post-transcriptional effects, we measured *SRFR1* transcript abundance. In *sum1-1* plants, *SRFR1* transcripts displayed only a slight reduction (∼0.8-fold), suggesting that reduced SRFR1 protein is mostly due to post-transcriptional effects (Figure 5B). Addition of the 26S proteasome inhibitor MG132 slightly improved HA-SRFR1 levels in *HA-SRFR1/sum1-1* plants. Furthermore, GFP-SUMO1 GG or AA variant co-expressed with HA-SRFR1 in *N. benthamiana* leaves caused an increase in HA-SRFR1 protein abundance in comparison with the GFP alone control (Figure 5C). Taken together, our results imply that *SUM1* role as a negative immune regulator may be via maintaining the steady-state levels of SRFR1.

Increased SA levels in *sum1-1* result in upregulated *SUM3* expression (van den Burg et al., 2010; Ingole et al., 2021b). To test whether reduced SRFR1 in *sum1-1* is a consequence of elevated *SUM3* expression, we generated *HA-SRFR1/sum3-1* plants. From the segregating population, plants expressing *HA-SRFR1* in *sum3-1* background showed significantly lower (∼2-fold) *SRFR1* transcript and protein levels than Col-0 (Figures 5A,B). Addition of MG132 did not improve HA-SRFR1 levels in *sum3-1*, implying that *SUM3* regulates *SRFR1* transcription (Figure 5A). Since we did not have the HA-SRFR1/*sum1-1 sum3-1* plants to investigate their cumulative effect on SRFR1 protein, we checked *SRFR1* transcript abundance in the *sum1-1 sum3-1* plants that we reported recently (Ingole et al., 2021b). *SRFR1* transcripts in *sum1-1 sum3-1* were intermediate between *sum1-1* and *sum3-1* levels, suggesting interplay among the SUMO isoforms on its steady-state expressions (Figure 5B). *SUM3* overexpression enhances basal defenses in *Arabidopsis* (van den Burg et al., 2010). With *SUM3* role in *SRFR1* transcriptions indicated from our results, we generated *HA-SRFR1* plants that overexpressed GFP-tagged SUMO3 (*GFP-SUM3*). These plants (*GFP-SUM3/HA-SRFR1* Lines#1 and #2) were homozygous for the *sum3-1* mutation. Anti-GFP immunoblot detected overexpressed GFP-SUMO3 in extracts from the transgenic lines (Supplementary Figure 9). We continued with Line#1 for further assays. Although downregulated *SRFR1* transcripts noted in *sum3-1* restored to Col-0 levels in the *GFP-SUM3* transgenic line, HA-SRFR1 protein were lower than the parental *HA-SRFR1* (Figures 5B–D). Unlike what was noted for *HA-SRFR1/sum3-1*, MG132 treatment stabilized HA-SRFR1 protein in the *GFP-SUM3* overexpressing plants, suggesting that as in *sum1-1*, the proteasome pathway was responsible for lower SRFR1 accumulations. Together with known *SUM3* overexpression enhancing basal defenses, our results connect these responses again to consequences of reduced SRFR1 levels (van den Burg et al., 2010).

To further substantiate this, we challenged *HA-SRFR1/sum1-1* or *HA-SRFR1/sum3-1* plants with virulent *PstDC3000* and evaluated SRFR1 protein changes (Figure 5E). Interestingly, lack of *SUM1* delayed SRFR1 restoration, and even at 24 hpi, HA-SRFR1 levels were barely detectable. Contrastingly, HA-SRFR1 recovery was rapid in *sum3-1* reaching native levels by 6 hpi. Accumulation of SUMO1/2-conjugates with *PstDC3000* challenge was dramatically lower in *sum3-1* and in agreement with our earlier observations (Ingole et al., 2021b; Figure 5F). Together, these data demonstrated intricate interplay between SUMO isoforms in basal defenses with *SUM1* essential for SRFR1 restitution at post-transcriptional level and *SUM3* modulating transcriptional efficiency as a part of feedback loop mechanism.

### Upregulated *SUM3* Maintains *SRFR1* Protein Levels in Autoimmune *esd4-2* Plants

**FIGURE 6:**
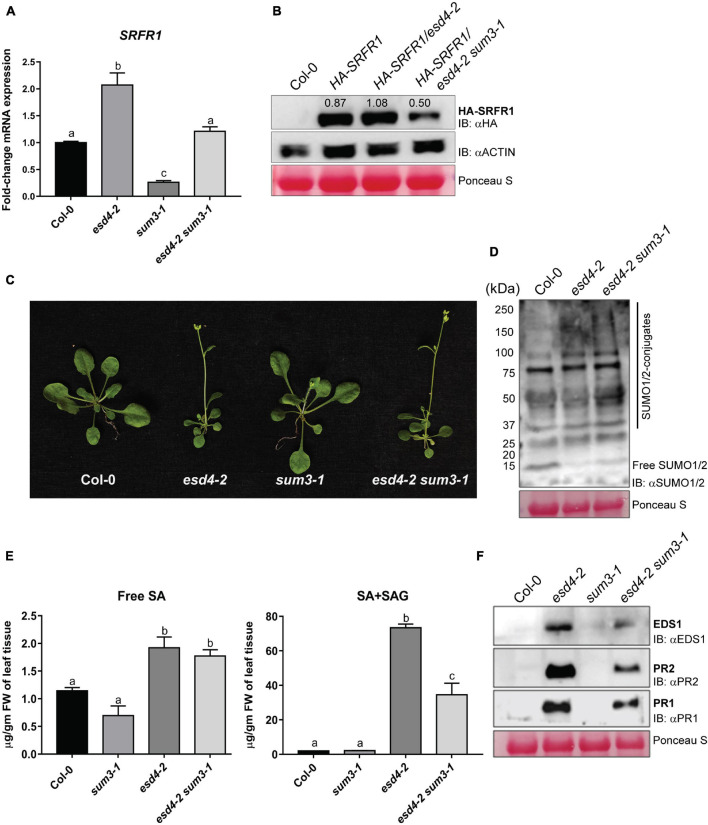
*SUM3* is partially responsible for increased defenses but not growth phenotype of *esd4-2*. **(A)** *SRFR1* transcript abundance in Col-0, *esd4-2*, *sum3-1*, and *esd4-2 sum3-1* plants. Expressions were compared with *MON1* levels and presented as fold-change relative to Col-0 (*n* = 3). Statistical significance is by *post hoc* Tukey’s test (*p* < 0.05) and shown by different letters. **(B)** HA-SRFR1 protein in wild-type (*HA-SRFR1*), *HA-SRFR1*/*esd4-2*, and *HA-SRFR1/esd4-2 sum3-1* background. Col-0 is the negative control for the immunoblot. **(C)** *esd4-2 sum3-1* plants resemble *esd4-2* growth deficiencies. Images are representative of 4-week-old plants of indicated genotypes. **(D)** Global SUMOylome enhancements in *esd4-2* and *esd4-2 sum3-1* plants compared with Col-0. **(E)** Free and total SA levels, and **(F)** accumulation of EDS1, PR2, or PR1 proteins in *esd4-2*, *sum3-1*, and *esd4-2 sum3-1* plants compared with Col-0. Protein blots were probed with anti-HA, anti-SUMO1/2, anti-EDS1, anti-PR1, or anti-PR2 antibodies, as indicated. Migration positions of SUMO1/2-conjugates, free SUMO1/2, or molecular weight standards (in kDa) are shown. Similar protein loadings are shown by Ponceau S staining. Numbers above the HA-SRFR1 bands in panel **(B)** are densitometric quantification values relative to actin expression levels in the same extracts.

The SUMO-protease ESD4 mutant (*esd4-2*) has increased SUMO1/2-conjugates, elevated SA, and enhanced basal defenses (Villajuana-Bonequi et al., 2014). We showed earlier that *ESD4* transcriptions are downregulated during *PstDC3000* infections (Ingole et al., 2021a). Therefore, with overall similarities to basal defense patterns, we investigated the fate of SRFR1 in *esd4-2*. *SRFR1* transcripts were significantly higher (∼2-fold) likely due to higher *SUM3* expressions in *esd4-2* (Figure 6A; Villajuana-Bonequi et al., 2014). To assess protein levels, we generated *HA-SRFR1/esd4-2* plants by crossing *HA-SRFR1/sum3-1* to *esd4-2*. In F2 plants with loss of *ESD4* but wild-type *SUM3*, SRFR1 protein levels were unchanged (Figure 6B). This contrast between SRFR1 transcriptional upregulations versus its unchanged protein levels in *esd4-2* plants once again indicated complex regulations on SRFR1 expressions by SUMOylome perturbations.

To directly determine whether *SUM3* contributed to this process, from the above cross, we identified *HA-SRFR1/esd4-2 sum3-1* plants in the F2 and F3 populations. These plants were genetically similar to *esd4-2 sum3-1* double mutant. Growth deficiencies including early bolting and enhanced global SUMO1/2-conjugates in *esd4-2* were not affected by *SUM3* loss (Figures 6C,D). Levels of free SUMO1/2, however, showed lower levels than in Col-0, suggesting that ESD4 SUMO-protease functions are essential for maintaining these pools of SUMOs. *sum3-1* reduced total but not free SA levels and also lowered the protein or transcript abundance of several positive defense-associated players (EDS1, PR1, or PR2) that were elevated in the *esd4-2* background (Figures 6E,F and Supplementary Figure 10A). Introducing *sum3-1* in *esd4-2* reduced *SRFR1* transcripts to Col-0 levels, whereas protein abundance was lower than that in *HA-SRFR1* or *HA-SRFR1/esd4-2* plants (Figures 6A,B). We accord this to transcriptional promotion of *SUM3* on *SRFR1* observed earlier. Overall, these data reiterated that relative levels of *SUMO1/3* modulate *SRFR1* expression.

Several TFs are SUMOylation targets (Miller et al., 2010). SUMOylome disturbances cause expression differences of several SUMOylation-associated genes (Ingole et al., 2021b). To evaluate this in *esd4-2* combination mutants, we performed quantitative real-time PCRs (qRT-PCRs). Expressions especially for *SIZ1* and *HPY2*, the two E3 ligases, were higher and *SUM3*-independent in *esd4-2* (Supplementary Figure 10C). *SUM1* transcripts remain unaffected in all plants tested (Supplementary Figure 10B). Taken together, these data reflected consequences of deficient ESD4 activity rather than increased SUMOylation efficiencies by upregulated *SIZ1*/*HPY2* or due to *SUM3* involvement in their expressions as the primary cause of elevated SUMO1/2-conjugates in *esd4-2* plants.

### Enhanced Basal Defenses in *srfr1-4* Are Partially *SUM3*-Dependent

Our results here and earlier suggested mutual expression influences between *SRFR1* and SUMOylation-associated genes (Ingole et al., 2021a). To evaluate this interplay in the context of enhanced immunity in *srfr1-4*, we attempted to generate *srfr1-4 sum1-1* or *srfr1-4 sum3-1* double mutants. Considering *SUM1* role as a negative immune regulator and *SUM3* as a positive immune regulator, we tested whether pathogenesis outcomes of *srfr1-4* are altered. From the F2/F3 segregating population of *sum1-1* crossed with *HA-SRFR1* plants used earlier, we screened for *srfr1-4 sum1-1* double mutants. Even after extensive screening, *srfr1-4 sum1-1* plants were not obtained, suggesting their embryonic lethality. It remains a possibility that developmental consequences that occur in *srfr1-4* are compounded by the loss of *SUM1*. Observed growth and immune enhancements that we reported recently in *sum1-1* support our speculation (Ingole et al., 2021b).

**FIGURE 7:**
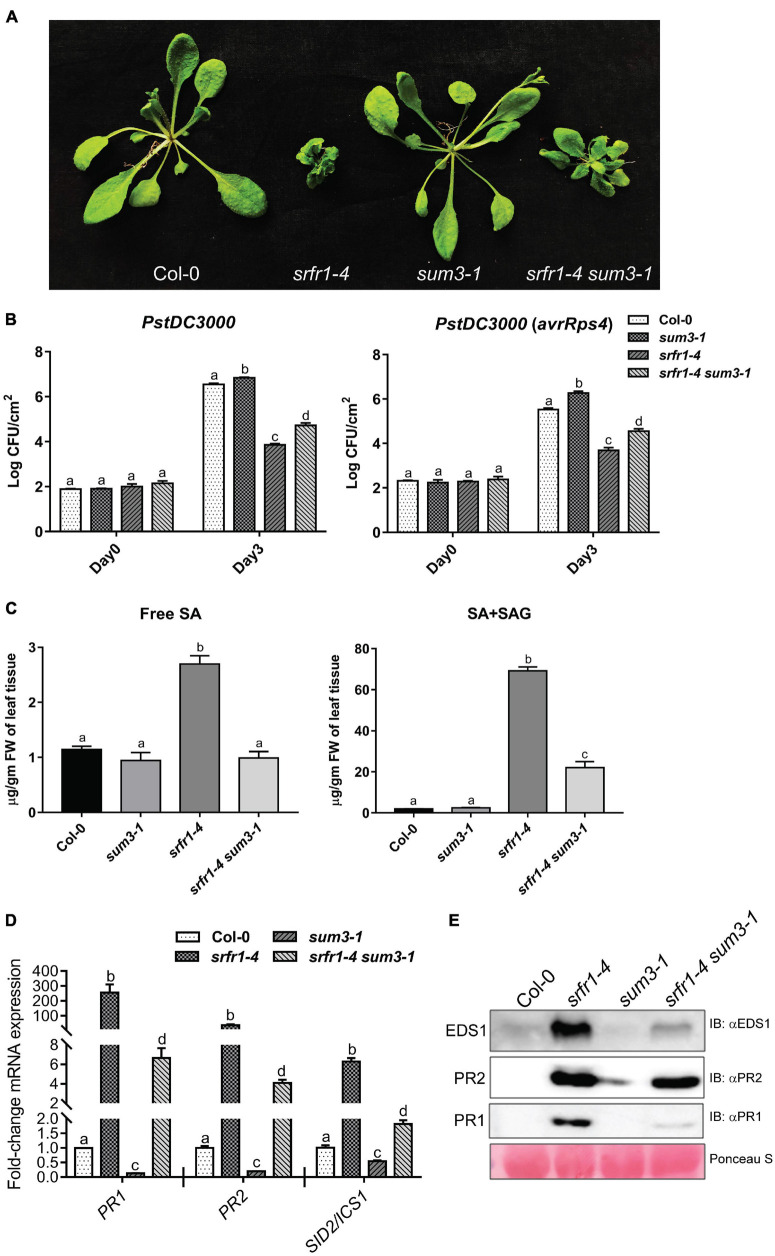
Enhanced defenses in *srfr1-4* are partially *SUM3*-dependent. **(A)** Growth phenotypes of 3- to 4-week-old plants of indicated genotypes. **(B)** Bacterial growth measurements with virulent *PstDC3000* or avirulent *PstDC3000* (*avrRps4*) strains; **(C)** free and total SA levels; **(D)** transcript abundance of *PR1*, *PR2*, or *SID2/ICS1*; and **(E)** protein levels of EDS1, PR1, or PR2 in Col-0, *srfr1-4*, *sum3-1*, or *srfr1-4 sum3-1* plants. Growth of bacterial strains at day 0 or day 3 post-challenge is shown. Statistical significance in transcript expression levels is via *post hoc* Tukey’s test (*p* < 0.05) and marked by different letters (*n* = 3).

We identified *srfr1-4 sum3-1* in the segregating populations of *sum3-1* crossed with *HA-SRFR1*. As observed for *srfr1-4 sid2-1* plants, *sum3-1* mutation does not restore growth defects of *srfr1-4* (Figure 7A). Enhanced SUMO1/2-conjugates were also unaffected by introducing *sum3-1* in *srfr1-4* (Figures 2B–D). Heightened expression of *SCE1* but not *SIZ1* in *srfr1-4* was abolished in *srfr1-4 sum3-1* plants, meaning that mis-regulations of some SUMOylation-associated genes in *srfr1-4* are *SUM3*-dependent. To compare defense responses, these plants were challenged with virulent *PstDC3000* or avirulent *PstDC3000* (*avrRps4*) strains. Surprisingly, unlike deficient immunity in *sum3-1* plants (Ingole et al., 2021b), bacterial accumulation for both strains in *srfr1-4 sum3-1* was lower than in Col-0 but higher than in *srfr1-4* (Figure 7B). Thus, *srfr1-4* mutation was epistatic to *sum3-1*. Free SA elevations were abolished, whereas total SA levels though are reduced than *srfr1-4* and remained significantly (∼20-fold) higher in *srfr1-4 sum3-1* to Col-0 (Figure 7C). Relative expression levels of *PR1*, *PR2*, and *SID2/ICS1* transcripts were also higher than those of Col-0 in *srfr1-4 sum3-1* but lower than in *srfr1-4* plants (Figure 7D). Similarly, protein levels of PR1, PR2, or EDS1 were also elevated than Col-0 but lower than *srfr1-4* in the double mutant (Figure 7E). These results indicated that upregulated defenses in *srfr1-4* only partially involved *SUM3* contributions. Together, our data suggested that SRFR1 dynamics primarily modulated defense-associated SUMOylome changes and immune amplitudes in partial *SUM3*-dependent and SA-dependent manner. Overall, with our investigations, here we reveal an intricate molecular crosstalk between SRFR1 role in SUMOylome homeostasis/adjustments during defense and counter-repercussions on SRFR1 expressions by the SUMOylation changes overall to modulate immune amplitudes.

## Discussion

Pleiotropic phenotypes in the context of host SUMOylome alterations have remained restricted to studies in mutants of SUMOylation pathway genes (Lee et al., 2007; van den Burg et al., 2010; Villajuana-Bonequi et al., 2014; Bailey et al., 2016). We first demonstrated the role of a negative immune regulator SRFR1 in maintenance of host SUMOylome homeostasis (Ingole et al., 2021a). That loss of *SRFR1* caused upregulated SUMOylation-promoting and downregulated SUMO protease expressions, a consequence mirrored in basal defenses, genetically placed SRFR1 as a transcriptional modulator of the host SUMOylome (Ingole et al., 2021a). Also, with our demonstration that *SUM1* transcripts are preferentially loaded onto polysomes in *srfr1-4* plants, we further expanded SRFR1 involvement also at the post-transcriptional level. Here, using *srfr1-4 sid2-1* or *srfr1-4 snc1-11* plants, we reaffirmed that similar to *srfr1-4 eds1-2*, elevated SA through *SID2/ICS1*, and *SNC1* upregulated expressions in *srfr1-4* are the principal cause of increased SUMO1/2-conjugates (Ingole et al., 2021a). Taken in the context of SUMO1/2-conjugation increase observed upon SA treatment, SRFR1 suppression of *SID2/ICS1* expressions likely maintains global SUMOylome and mis-priming of immunity (Kim et al., 2010; Bhattacharjee et al., 2011; Bailey et al., 2016; Ingole et al., 2021a).

SA targets SUMO proteases OTS1/2 for proteasome-mediated degradation, thus enhancing SUMO1/2-conjugate levels (Bailey et al., 2016). We show here that SRFR1 undergoes transient instability when basal defenses are induced upon *PstDC3000* exposure. The reduction in SRFR1 correlates with subsequent increase in SA levels and SUMO1/2-conjugate enhancements. Considering SA degrades OTS1/2, SRFR1 changes via downstream SA increase may drive the condemned fates of OTS1/2, presenting another example of its host SUMOylome adjustment role at the post-transcriptional level (Bailey et al., 2016). Recuperation of SRFR1 levels at latter time points of basal defense elicitation perhaps is indicative of fine-tuning of immune amplitudes, preventing overshoots or pleiotropic consequences like in *srfr1-4* or in the SUMOylation-perturbed mutants such as *siz1-2* or *esd4-2* (Lee et al., 2007; Kim et al., 2010; Villajuana-Bonequi et al., 2014).

SUMOylome changes in turn regulate SRFR1 expressions. Firstly, in *sum1-1* plants, unchanged *SRFR1* transcripts but reduced protein levels are stabilized by the proteasome inhibitor MG132. Secondly, increased SRFR1 protein accumulation is detected by transient overexpression of *SUM1* in *N. benthamiana* leaves. Thirdly, SRFR1 restoration is deficient in *sum1-1* plants when challenged with *PstDC3000*. Last but not the least, gradual restoration of SRFR1 protein levels parallels progressive increase in SUMO1/2-conjugates during *PstDC3000* challenges. Overall, these results suggest that *SUM1* affects SRFR1 at a post-transcriptional level, possibly regulating protein turnover. Unlike *sum1-1*, reduced SRFR1 protein in *sum3-1* matches its lower transcript levels. Taking into consideration our recent data that *SUM3* potentiates SUMO1/2-conjugation efficiencies and in *sum3-1* plants endogenous as well as defense-induced increase in SUMO1/2-conjugates are deficient, lower SRFR1 levels in this mutant likely also incorporate additive effects from this intersection (Ingole et al., 2021b). Thus, *sum3-1* partially mimics *sum1-1* consequences in destabilizing SRFR1. This notion is supported by our observation that MG132 treatment improves SRFR1 protein stability to a modest extent in *sum3-1*. Immune responses, however, remain deficient in *sum3-1* likely because of functional inadequacies in the SA-signaling sector through NPR1, a known exclusive SUMO3-substrate (Saleh et al., 2015).

*SUM3* expression is induced transiently at early time points of SA application (van den Burg et al., 2010). Increased SA levels and concomitant upregulation of *SRFR1* transcripts during basal defenses noted in our data imply transcriptional contributions of *SUM3* as we suggested above. However, SRFR1 instability also noted at same time points leads us to speculate that SUMO3, antagonistic to SUMO1, may negatively affect SRFR1 protein accumulation upon SUMOylation. Our data that SRFR1 is unstable in *SUM3* overexpressing transgenic lines are in accordance with this hypothesis. Presence of multiple predicted SUMOylation motifs and *in vitro* SUMOylation by SUMO3 more prominent than for SUMO1, indeed, present SRFR1 as a candidate whose functions/stabilities may in turn be affected by host SUMOylome/SUMO isoform ratio changes. Stimulus-driven SUMO isoform switches that affect the stability of a substrate have been widely documented in animal systems (Meulmeester et al., 2008; Zhu et al., 2008, 2009). The mammalian GTPase activating protein RanGAP1, although is equally modified by SUMO1/2/3 *in vitro*, conjugation *in vivo* to SUMO1, but not SUMO2, imparts more stability from isopeptidases (Zhu et al., 2009). Similarly, HDAC1 is targeted for degradation upon SUMOylation by SUMO1, but not SUMO2 (Citro et al., 2013). Reduced SRFR1 levels in *sum1-1* plants can therefore be also attributed to increased *SUM3* expressions (Ingole et al., 2021a). However, that the same is not noted for *esd4-2* plants with elevated *SUM3* levels indicates that ESD4 functions are necessary for this process. Indeed, involvement of a SUMO-protease has been reported recently for stimulus-dependent SUMO paralog switching and its impact on the substrate stability (Fasci et al., 2015).

**FIGURE 8:**
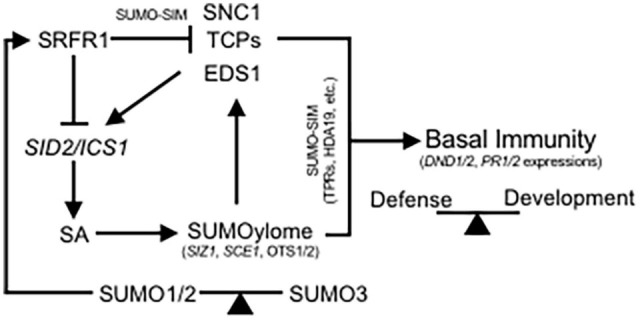
Simplified schematic representation of crosstalk between SRFR1 and SUMOylome maintenance in defense-developmental balance. Under homeostasis, SRFR1 prevents SUMOylome perturbations via its suppression of *SID2/ICS1* expression that drive SA responses. This mode of negative regulation incorporates transcriptional suppression of SUMO-conjugases (such as *SIZ1* and *SCE1*, among others), *SUM3*, and protein stabilities of SUMO proteases OTS1/2. Basal SUMOylome adjustments in turn reciprocates on SRFR1 function at multiple levels. As SUMOylation and/or SUMO-binding candidates, intermolecular SUMO-SIM-type associations of SRFR1 with SNC1, TCPs, and EDS1 prevent mis-primed immune activations. Similar nature of association occurring between these immune players and their downstream targets (TPRs and HDA19) regulates expression of defense-responsive genes such as *DND1/2* and *PR1/2*, among others. SUMO1/2 or SUMO3 isoforms with their respective roles as negative or positive immune modulators balance the association stoichiometry through SRFR1 expression/stability. During a pathogen threat, transient reduction in SRFR1 initiates SUMOylome perturbations and activates defense players to execute defense signaling. A feedback signaling loop also initiated restoration of SRFR1 levels to avoid development penalties of constitutive immunity.

Bioinformatics predictions of SIMs in SRFR1 taken together with non-covalent binding of multiple SUMO isoforms also present another mode by which a change in SUMO homeostasis may impact SRFR1 activities. Our observation that SRFR1 interaction with EDS1^K478R^, a speculated SUMOylation-deficient version, is abolished indicates biological implication of SRFR1 SIMs in interaction with positive defense regulators. The placement of a predicted SIMs (I^39^LDIC^43^) in the first TPR motif of SRFR1 (residues 39–72, TPRpred, toolkit.tuebingen.mpg.de), a well-known platform for protein–protein interaction, introduces encouraging direction to pursue further. Precise mutagenesis of this and other predicted SIMs followed by interaction analysis with EDS1 or other known interactors (such as RPS4/6, or SNC1) is needed to be tested *in vivo* (Kim et al., 2010; Bhattacharjee et al., 2011). With our assays, here, we are, however, unable to convincingly demonstrate EDS1 as an *in vivo* SUMOylation candidate. Since only a minor pool of EDS1 interacts with SRFR1, *in planta* identification of SUMOylated EDS1 may prove challenging (Bhattacharjee et al., 2011). SNC1 or TCP8/14/15 also contains multiple predicted SUMOylation motifs. Although SUMOylated SNC1 or TCPs has been detected *in vivo* or *in vitro*, respectively, their functional relevance remains untested (Gou et al., 2017; Mazur et al., 2017). Consolidated SUMO1/3 SUMOylome changes have strong potential to interfere with SRFR1 properties at multiple levels and modulate immune signaling. A mechanistic implication into this is presented in the schematic (Figure 8).

In summary, our results here provide a unique molecular basis into SRFR1 role in the SUMO-immune balances. With regulatory influences on expression of several SUMOylation-associated genes countered by SRFR1’s own stability determined by SUMO isoform crosstalk, we highlight its mediation of immune response amplitudes in plants (Ingole et al., 2021a). In addition to positive and negative immune regulators that are directly affected, SUMOylation efficiencies are also modulated by SUMO changes. This self-regulation is evident from the observation that SIZ1, SCE1, and ESD4 are themselves substrates for SUMOylation, and depending on the SUMO isoform they associate with, their localization or specificities are affected (Miller et al., 2010; Mazur et al., 2017). For example, SUMO1 bound to SCE1 SIM localizes the ternary complex (SUMO-SCE1-SIZ1) to nuclear bodies, whereas SUMO3 binding partitions it as nucleocytoplasmic. Overall, defense amplitudes therefore rely on strict harmony between SUMOylome modulators such as SRFR1, (de)SUMOylation efficiencies, localization of SUMOylation-machineries, and selection of substrates (Ingole et al., 2021a). Comprehensive elucidation of these events requires not only qualitative (i.e., which SUMO isoform-modified) but also quantitative (ratio of SUMOylated versus non-SUMOylated) evaluation of a host SUMOylome adaptation during immunity. While SUMO1/2-modified protein list is ever-increasing, SUMO3-targets remain grossly underrepresented. Development of an efficient SUMO3-enrichment system, on the similar theme to Miller et al. (2010), is therefore a necessity. Equally important is the characterization of protein–protein interactome changes that are defined by the SUMO-SIM nature. Lastly, functional intersection among SUMO-machineries, especially the SUMO-ligases/proteases, recently identified DeSIs (de-SUMOylating isopeptidases) (Orosa et al., 2018), STUbLs (SUMO-targeted ubiquitin ligases; Elrouby et al., 2013), and PIALs (E4-type SUMO ligases) (Tomanov et al., 2014), and their cognate substrates are warranted to decipher the net impact on immune signaling. Toward this endeavor, we present SRFR1 role in SUMOylome regulations and the *srfr1-4* mutant as a promising system to pursue these investigations.

## Data Availability Statement

The datasets presented in this study can be found in online repositories. The names of the repository/repositories and accession number(s) can be found below: PRIDE Archive, accession no: PXD026117.

## Author Contributions

SB and WG conceived the research. MK, KI, SR, and SB designed the research. SR generated T91R SUMO1 clone. MK and KI generated other clones and plants used here and performed the experiments. MK, KI, and SB analyzed the data. MK, WG, and SB wrote the manuscript. All authors contributed to the article and approved the submitted version.

## Conflict of Interest

The authors declare that the research was conducted in the absence of any commercial or financial relationships that could be construed as a potential conflict of interest.

## Publisher’s Note

All claims expressed in this article are solely those of the authors and do not necessarily represent those of their affiliated organizations, or those of the publisher, the editors and the reviewers. Any product that may be evaluated in this article, or claim that may be made by its manufacturer, is not guaranteed or endorsed by the publisher.

## Footnotes

1
www.NCBI.nlm.NIH.gov/RefSeq/

## Supplementary Material

Supplementary Figure 1 *snc1-11* abolishes enhanced SUMO1/2-conjugates and restores elevated SA to Col-0 levels in *srfr1-4*.

Supplementary Figure 2 MYB30 is SUMOylated in the *E. coli* SUMOylation reconstitution system by the T91R variant of SUMO1.

Supplementary Figure 3 A consensus-type and a non-conventional lysine residue in SRFR1 is SUMOylated by SUMO1 in the *E. coli* SUMOylation reconstitution system.

Supplementary Figure 4 Growth defects of *srfr1-4* is abolished by the expression of HA-SRFR1^K325R^, HA-SRFR1^K427R^, or HA-SRFR1^K325R+K427R^ variant.

Supplementary Figure 5 EDS1 does not interact with SUMO1 AA or SUMO3 GG/AA.

Supplementary Figure 6 EDS1 SUMOylation is not detected in the *E. coli* SUMOylation reconstitution system.

Supplementary Figure 7 Enrichment of SUMO1-conjugates from SA-treated plants detect possible SUMOylated EDS1.

Supplementary Figure 8 Over-expression of SUMO1 or SUMO3 *in planta* does not detect SUMOylated EDS1.

Supplementary Figure 9 *GFP-SUM3* overexpressing plants show detectable levels of GFP-SUMO3 proteins.

Supplementary Figure 10 *PR1*, *PR2*, but not *SIZ1* or *HPY2* upregulation in *esd4-2* plants is *SUM3*-dependent.

Supplementary Table 1 List of oligonucleotide primers.
