# Supplementary material for: Global SUMOylome Adjustments in Basal Defenses of Arabidopsis thaliana Involve Complex Interplay Between SMALL-UBIQUITIN LIKE MODIFIERs and the Negative Immune Regulator SUPPRESSOR OF rps4-RLD1
Source: Front Cell Dev Biol. 2021 Sep 30;9:680760. doi: 10.3389/fcell.2021.680760 (PMC8514785; doi:10.3389/fcell.2021.680760)
Supplement: Supplementary Figure 1 — snc1-11 abolishes enhanced SUMO1/2-conjugates and restores elevated SA to Col-0 levels in srfr1-4. [file Data_Sheet_1.PDF]

Supplemental Figures

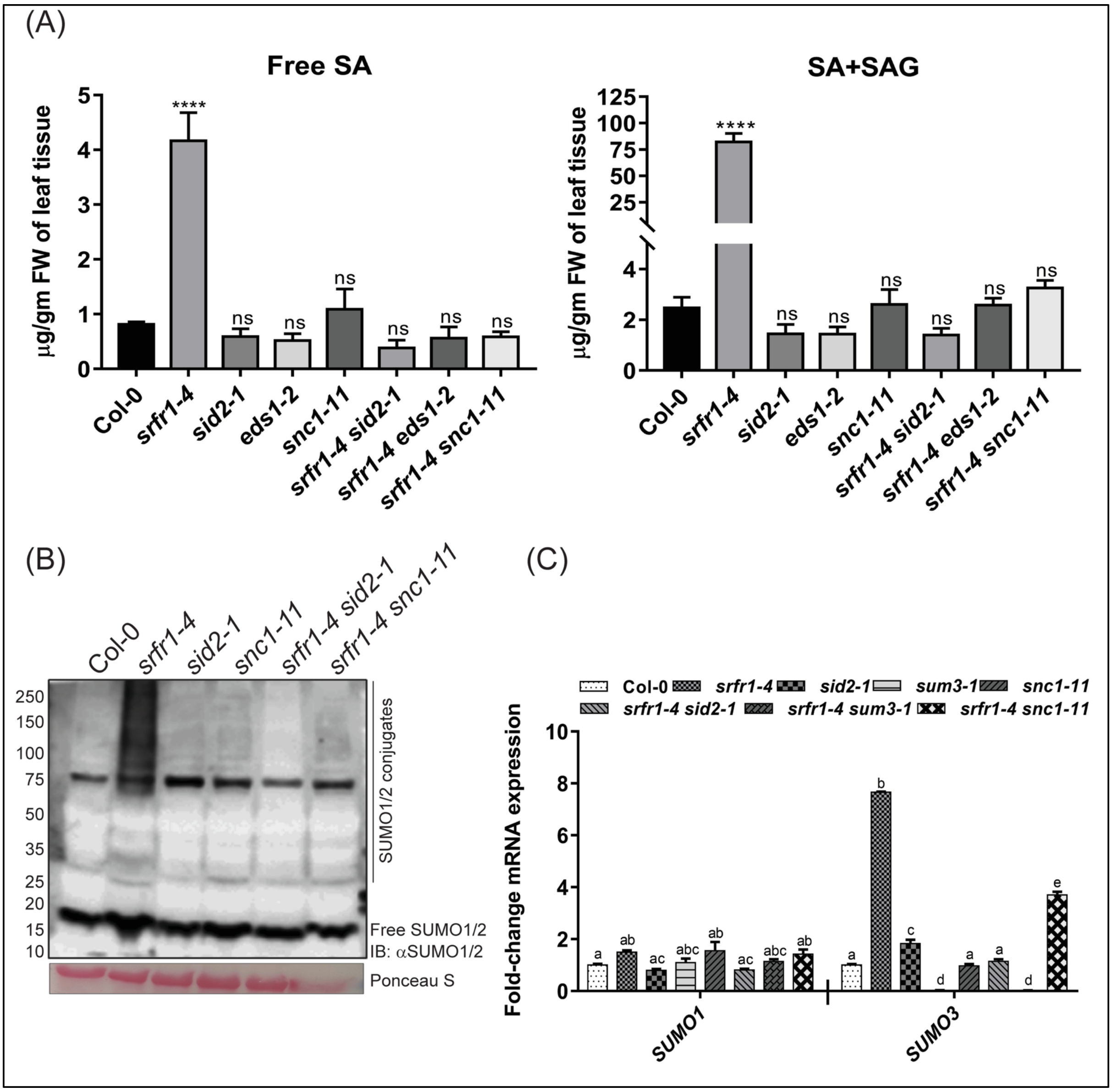

Figure S1. *snc1-11* abolishes enhanced SUMO1/2-conjugates and restores elevated SA to Col-0 levels in *srfr1-4*. (A) Total (SA+SAG) and free SA levels in Col-0, *srfr1-4*, *sid2-1*, *eds1-2*, s*nc1-11*, *srfr1-4 sid2-1*, *srfr1-4 eds1-2*, and *srfr1-4 snc1-11* plants. Values are presented as mean ± SD of at least three biological replicates (n=3). Statistical analysis is according to pairwise Student's *t*-test to Col-0 (*****p*<0.0001 and ns=not significant). **(B)** Anti-SUMO1/2 immunoblot on total protein extracts from indicated plants. Relative positions of SUMO1/2-SUMOylated proteins, free SUMO1/2, and molecular weight standards (in kDa) are shown. Ponceau S staining indicates loading controls. **(C)** Relative expression of *SUM1* and *SUM3* in indicated plant genotypes. Values are relative to internal control *MON1* gene expressions and represent mean ± SD (n=3). Different alphabets represent statistical differences according to post-hoc Tukey's test (*p*<0.05).

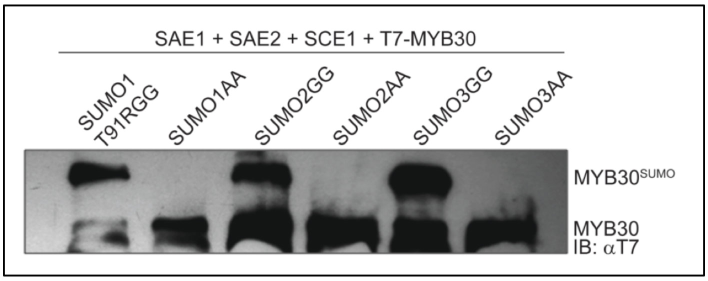

Figure S2. MYB30 is SUMOylated in the *E. coli* SUMOylation reconstitution system by the T91R variant of SUMO1. Anti-T7 immunoblot on extracts co-expressing T7-MYB30 with SUMO1 T91R GG/AA, SUMO2 GG/AA, or SUMO3 GG/AA forms. $MYB30^{SUMO}$ or MYB30 indicate migration positions of SUMOylated or non-SUMOylated MYB30, respectively.

%Cov(95)	Accession	Protein Name	Peptides(95%)
46.2	gi|186517170	suppressor of RPS4-RLD 1 [Arabidopsis thaliana]	65
45.11	gi|4468812	putative protein [Arabidopsis thaliana]	64
49.14	gi|42570865	SUMO-activating enzyme subunit 2 [Arabidopsis thaliana]	28
55.04	gi|30681414	SUMO-activating enzyme subunit 2 [Arabidopsis thaliana]	28
54.78	gi|334184353	SUMO-activating enzyme subunit 2 [Arabidopsis thaliana]	28
46.27	gi|28467490	putative ubiquitin activating enzyme [Arabidopsis thaliana]	25
76.92	gi|1707372	ubiquitin-like protein [Arabidopsis thaliana]	35
80	gi|15236885	small ubiquitin-related modifier 1 [Arabidopsis thaliana]	35
56.88	gi|15230881	SUMO-conjugating enzyme SCE1 [Arabidopsis thaliana]	12
26.09	gi|18416454	SUMO-activating enzyme subunit 1A [Arabidopsis thaliana]	7
26.09	gi|1032282704	SAE1A [Arabidopsis thaliana]	7

Protein	Peptide detected	Modification
SRFR1 @K229	CGSQINGYYESCKPCNGSDLHDNLAESSDR	GlyGly(K)@13
SRFR1 @K325	VLKEEPTYPEALIGR	GlyGly(K)@3

Figure S3. A consensus-type and a non-conventional lysine residue in SRFR1 is SUMOylated by SUMO1 in the *E. coli* SUMOylation reconstitution system. Peptide enrichments in LC-MS/MS analysis of SUMO1-SUMOylated proteins in eluted samples (*top table*). Peptide sequences were categorized using the *Arabidopsis thaliana* protein database (www.NCBI.nlm.NIH.gov/RefSeq/). SRFR1 and SUMO1 enrichments are highlighted in red. Proteins ≥95% confidence level were considered. Table shown below indicates the position of SUMOylated lysine residues (in red) identified in SRFR1 peptides.

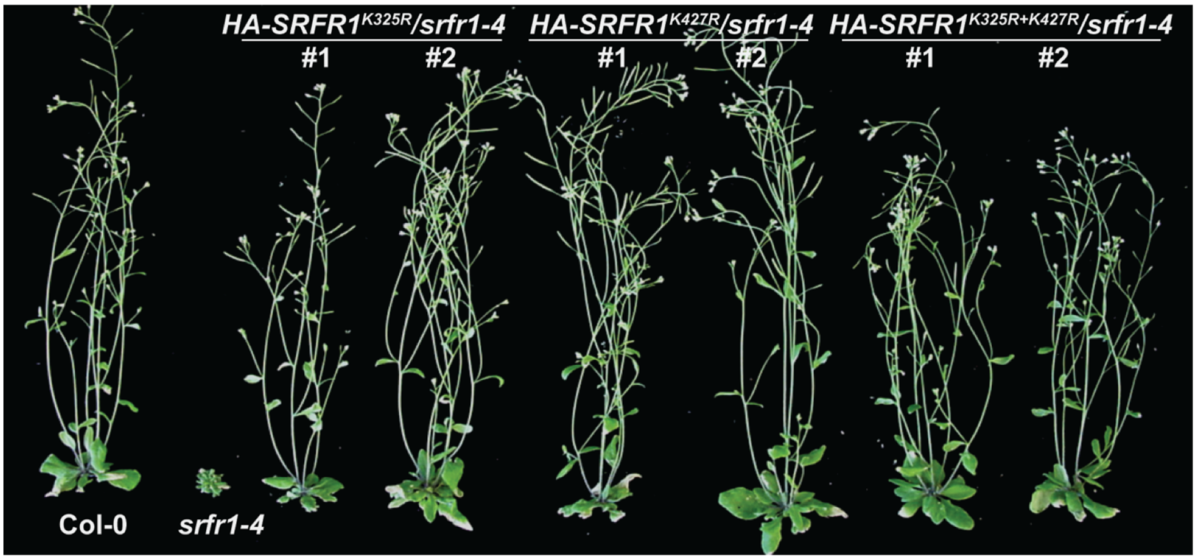

Figure S4. Growth defects of *srfr1-4* is abolished by the expression of HA-SRFR1^{K325R}, HA-SRFR1^{K427R}, or HA-SRFR1$^{K325R+K427R}$ variant. Two independent transgenic lines generated for each *HA-SRFR1* variant were imaged at 6 week-old growth stage. Two independent transgenic lines of each type, Col-0, and *srfr1-4* are shown.

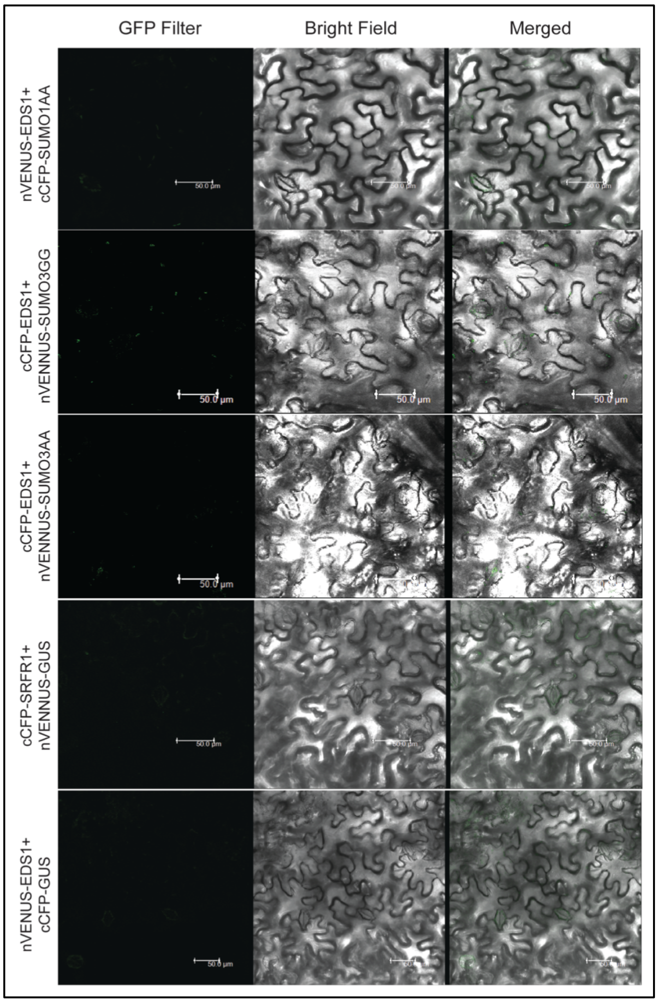

Figure S5. EDS1 does not interact with SUMO1 AA or SUMO3 GG/AA. Panels showing lack of detectable fluorescence between EDS1 and selective SUMO1 or SUMO3 clones (*top 3 rows*). Bottom two rows are the BiFC control combinations for SRFR1 or EDS1 with GUS.

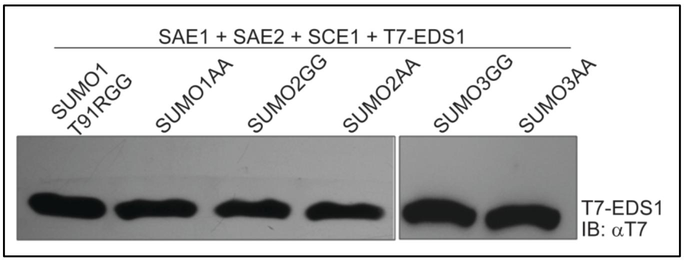

Figure S6. EDS1 SUMOylation is not detected in the *E. coli* SUMOylation reconstitution system. Extracts from cell expressing T7-EDS1 with SUMO1 T91R, SUMO2, or SUMO3 GG/AA forms in the presence of other SUMOylation machineries were immunoblotted with anti-T7 antibodies. Migration position of T7-EDS1 is marked.

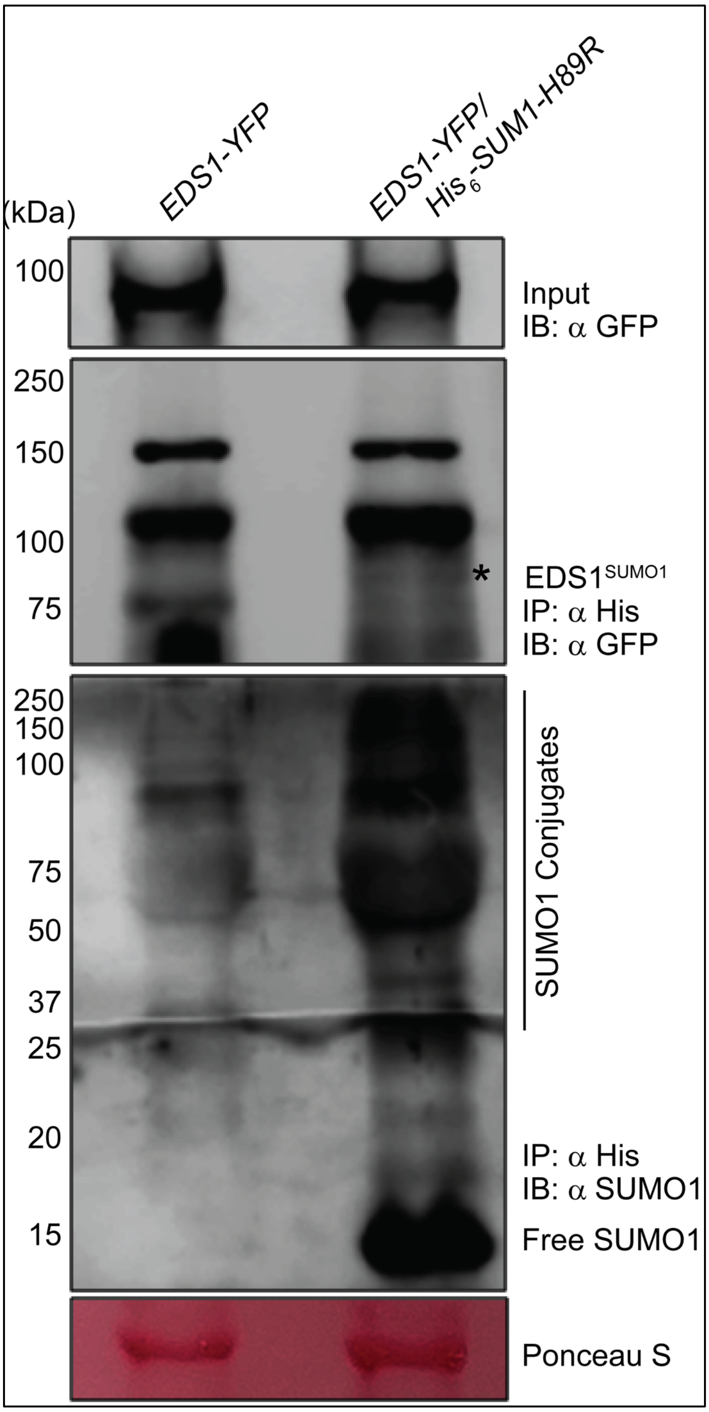

Figure S7. Enrichment of SUMO1-conjugates from SA-treated plants detect possible SUMOylated EDS1. Plants expressing EDS1-YFP alone or with His_6-SUM1 H89R were treated with SA followed by enrichment of His_6-SUMO1 conjugates under denaturing conditions with Ni^{2+} matrix. Bound proteins were probed with anti-GFP or anti-SUMO1 antibodies. Asterisk indicates the position of predicted SUMOylated EDS1 (*second panel*). Enrichment of SUMO1-conjugates are shown (*third panel*). EDS1-YFP expression in the input fractions are also indicated (*top panel*). Comparable protein loading for the input fractions are shown with Ponceau S staining. The migration position of molecular weight standards (in kDa) are marked.

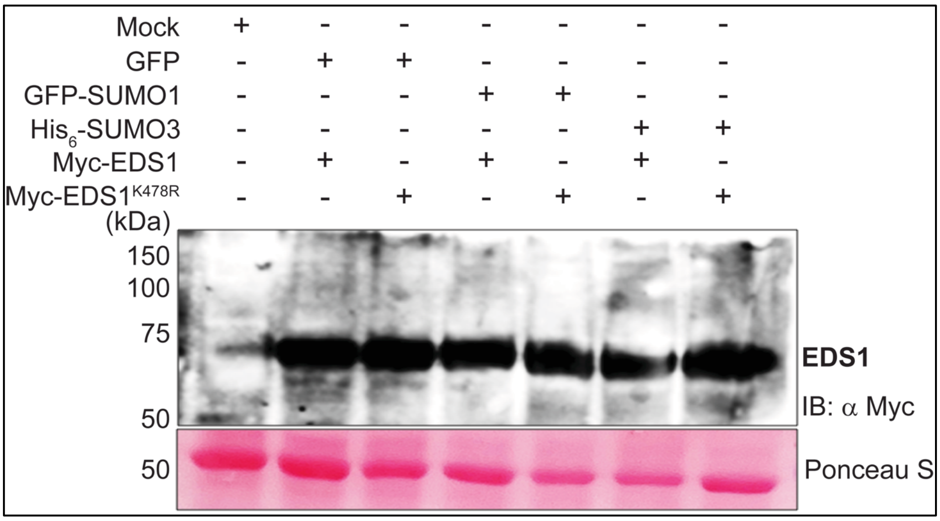

Figure S8. Over-expression of SUMO1 or SUMO3 *in planta* does not detect SUMOylated EDS1. Myc-EDS1 (wild-type or K478R version) was co-expressed with GFP alone, GFP-SUMO1 or His_6-SUMO3 in *N. benthamiana* leaves. Total protein extracts were immunoblotted with anti-Myc antibodies. Migration position of molecular weight standards (in kDa) and Myc-EDS1 is indicated. Protein loading is indicated by Ponceau S-staining of the membrane.

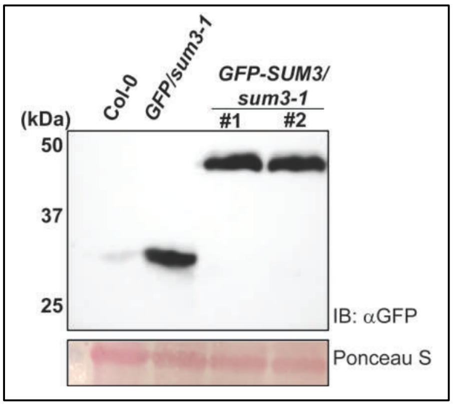

Figure S9. ***GFP-SUM3*** **overexpressing plants show detectable levels of GFP-SUMO3 proteins.** Total extracts from *GFP/sum3-1* Line#1 and Line#2 and *sum3-1* plants expressing GFP alone (*GFP-SUM3/sum3-1*) were immunoblotted with anti-GFP antibodies. Migration position of molecular weight standards (in kDa) are indicated. Comparable protein loading is indicated by Ponceau S staining of the membrane.

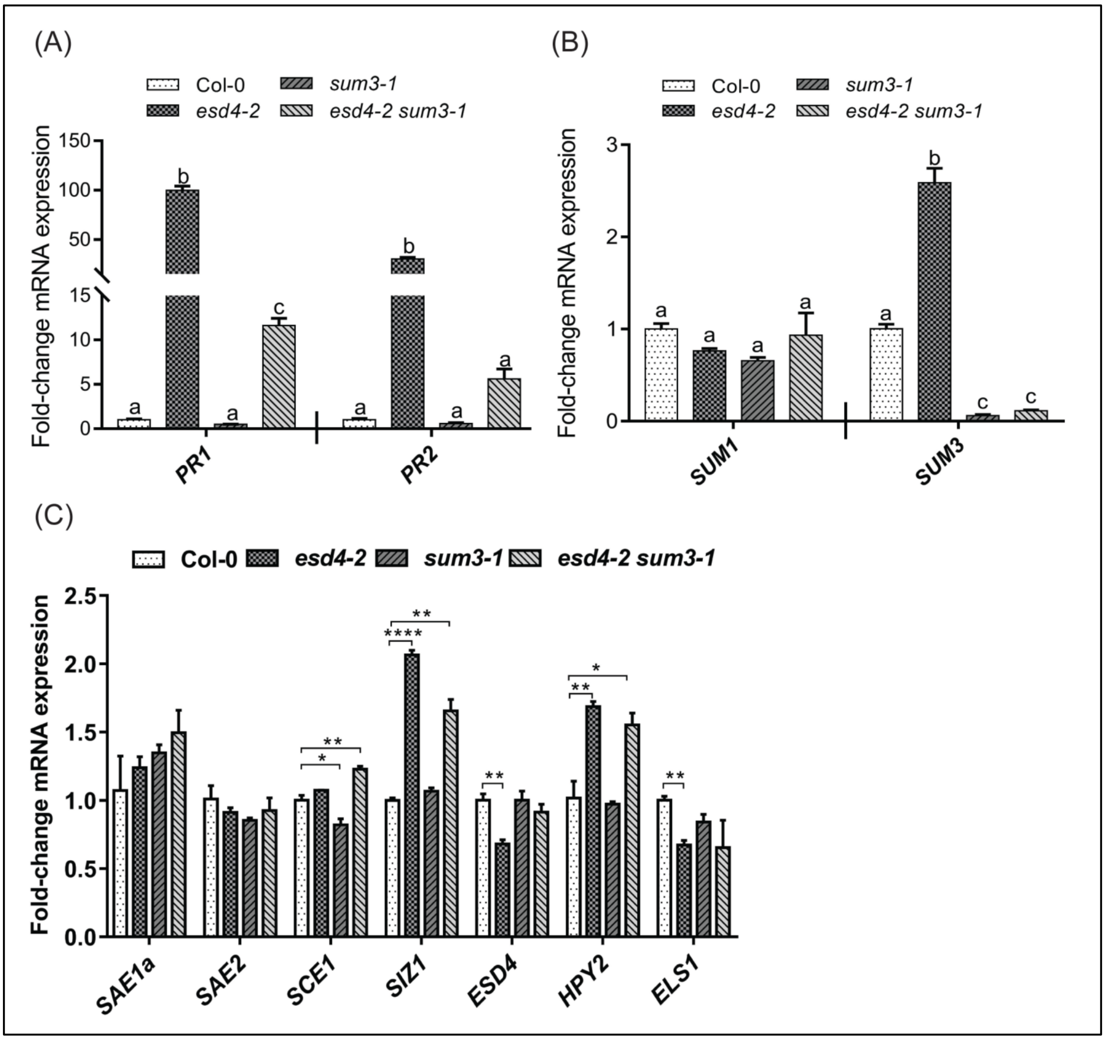

Figure S10. *PR1*, *PR2*, but not *SIZ1* or *HPY2* upregulation in *esd4-2* plants is *SUM3*-dependent. Relative abundance of **(A)** *PR1*, *PR2*, **(B)** *SAE1a*, *SAE2*, *SCE1*, *SIZ1*, *ESD4*, *HPY2*, *ELS1*, **(C)** *SUM1* and *SUM3* transcripts in the indicated plant genotypes. Expressions are relative to *MON1* levels with data showing mean ± SD (n=3). Values are reported as fold-change relative to Col-0 levels. Different alphabets are according to post-hoc Tukey's test (p<0.05) for A and B. For C, statistical significance is according to Student's t-test (*=p<0.05; **=p<0.01; ***=p<0.001, ns= not significant).

Supplementary Table 1: List of oligonucleotide primers

S. No	Primer Names	Primer Sequence (5' to 3')	Purpose (Ref)
1.	*SUM1 T91R* For	GATGCGATGCTCCATCAGCGTGGT GGC	Generating SUMO1 T91R (*This study*)
2.	*SUM1 T91R* Rev	GCCACCACGCTGATGGAGCATCGC	Generating SUMO1 T91R (*This study*)
3.	*EDS1* K478R For	TTAAGGAACGAAGACACAGGGCCG	Generating EDS1 K478R (*This study*)
4.	*EDS1* K478R Rev	CTTCGTTCCTTAAATGTCGATGGT	Generating EDS1 K478R (*This study*)
5.	*p*DONR201 For	GTATTACCGCTAGCCAGGAAGAGT	*p*DONR201 screening primer (*This study*)
6.	*p*DONR201 Rev	GCGCATCAACAATATTTTCACCTG	*p*DONR201 screening primer (*This study*)
7.	*SRFR1 K325R* For	GCAGGTGCTGAGAGAAGAACCAAC TTAC	Generating SRFR1 K325R (*This study*)
8.	*SRFR1 K325R* Rev	GTAAGTTGGTTCTTCTCTCAGCACC TGC	Generating SRFR1 K325R (*This study*)
9.	*SRFR1 K427R* For	TATGTCTGAGGCAAGAGAAAGATA AC	Generating SRFR1 K427R (*This study*)
10.	*SRFR1 K427R* Rev	GTTATCTTTCTCTTGCCTCAGACAT A	Generating SRFR1 K427R (*This study*)
11.	*SRFR1* For	CTGGATATGCCTCACTAGAAG	*SRFR1* qRT-PCR (*Kim et al., 2010*)
12.	*SRFR1* Rev	CACTGGGTCACAAGGCTCTG	*SRFR1* qRT-PCR (*Kim et al., 2010*)
13.	*MON1* For	AACTCTATGCAGCATTTGATCCACT	*MON1* qRT-PCR (*Kim et al., 2010*)
14.	*MON1* Rev	TGATTGCATATCTTTATCGCCATC	*MON1* qRT-PCR (*Kim et al., 2010*)
15.	*PR1* For	CCCTCGAAAG CTCAAGATAG	*PR1* qRT-PCR (*Kim et al., 2010*)
16.	*PR1* Rev	GTTCACATAATTCCCACGAGG	*PR1* qRT-PCR (*Kim et al., 2010*)

17.	*PR2* For	TCAAGGAAGGTTCAGGGATG	*PR1* qRT-PCR (*Ingole et al., 2021*)
18.	*PR2* Rev	TTCACGAGCAAGGGAGATTG	*PR1* qRT-PCR (*Ingole et al., 2021*)
19.	*SID2/ICS1* For	GCAACAACATCTCTACAGGCG	*SID2/ICS1* qRT-PCR (*Kim et al., 2010*)
20.	*SID2/ICS1* Rev	AGAACCCCTTATCCCCCATA	*SID2/ICS1* qRT-PCR (*Kim et al., 2010*)
21.	*SIZ1* For	AACAGGGAAAGAAGCAGGAA	*SIZ1* qRT-PCR (*Ingole et al., 2021*)
22.	*SIZ1* Rev	GGCAGCTTGTTCATCAGAAA	*SIZ1* qRT-PCR (*Ingole et al., 2021*)
23.	*HPY2* For	CTACACCTTCCTCAGTGCCA	*SIZ1* qRT-PCR (*Ingole et al., 2021*)
24.	*HPY2* Rev	AACATTCCAAACTGCTTCCC	*SIZ1* qRT-PCR (*Ingole et al., 2021*)
25.	*SUM1* For	CTTGTTTGATGGGCGTCGTC	*SUM1* qRT-PCR (*Ingole et al., 2021*)
26.	*SUM1* Rev	CAGTCTGATGGAGCATCGCA	*SUM1* qRT-PCR (*Ingole et al., 2021*)
27.	*SUM3* For	CGAGCAAATCAGCGTCAGTG	*SUM3* qRT-PCR (*Ingole et al., 2021*)
28.	*SUM3* Rev	ACACACACGATAACCGACCA	*SUM3* qRT-PCR (*Ingole et al., 2021*)
29.	*SAE1a* For	ATTCCTCGGAGAACAGCAAA	*SAE1a* qRT-PCR (*Ingole et al., 2021*)
30.	*SAE1a* Rev	ACGCCCTTCACTCTCTTCAA	*SAE1a* qRT-PCR (*Ingole et al., 2021*)
31.	*SAE2* For	ACAGCCTTTTTGAAGCGAAA	*SAE2* qRT-PCR (*Ingole et al., 2021*)
32.	*SAE2* Rev	ATAATGGCGTTGGTCGTAGC	*SAE2* qRT-PCR (*Ingole et al., 2021*)
33.	*SCE1* For	AATGGTGTGGCATTGCACTA	*SCE1* qRT-PCR (*Ingole et al., 2021*)
34.	*SCE1* Rev	TCCTCACTGAAGTGCATCGT	*SCE1* qRT-PCR (*Ingole et al., 2021*)

35.	*ESD4* For	TGATGCTGGATTCGTTGTCG	*ESD4* qRT-PCR (*Ingole et al., 2021*)
36.	*ESD4* Rev	TCTCCGCACTTTGCATAAGC	*ESD4* qRT-PCR (*Ingole et al., 2021*)
37.	*ELS1* For	TGGATGGTTACCACAAACGG	*ELS1* qRT-PCR (*Ingole et al., 2021*)
38.	*ELS1* Rev	ATGAACTTGTCTCCGCAACG	*ELS1* qRT-PCR (*Ingole et al., 2021*)
39.	*sum1-1* LP	TTTCGTGTAGCTGCGATTAGG	Genotyping of *sum1-1* (*Ingole et al., 2021*)
40.	*sum1-1* RP	TTATCTTTGCTCGCCATTAGC	Genotyping of *sum1-1* (*Ingole et al., 2021*)
41.	*sum3-1* dspm1	CTTATTTCAGTAAGAGTGTGGGGTT TTGG	Genotyping of *sum3-1* (*van den Burg et al., 2010*)
42.	*LB3*	TAGCATCTGAATTTCATAACCAATC TCGATACAC	*SAIL* collection T-DNA border primer
43.	*SALK_LB1.3*	ATTTTGCCGATTTCGGAAC	*SALK* collection T-DNA border primer
44.	*Sm108F*	TTCTTCATGCAGGGGAGGAG	Genotyping of *sid2-1* (*Ingole et al., 2021*)
45.	*Sm30F*	CAACCACCTGGTGCACCAGC	Genotyping of *sid2-1* (*Ingole et al., 2021*)
46.	*L1849R*	AAGCAAAATGTTTGAGTCAGCA	Genotyping of *sid2-1* (*Ingole et al., 2021*)
47.	37460-18	TCTCCACTGTACTAATTTCCCT	Genotyping of *srfr1-4* (*Kim et al., 2020*)
48.	37460-R	ACTAATTCCGCAACGTGCCT	Genotyping of *sid2-1* (*Kim et al., 2010*)
49.	*esd4-2* LP	TTCATGGGATACAGAAGCCAG	Genotyping of *esd4-2* (*This study*)
50.	*esd4-2* RP	CTTATGCAAAGTGCGGAGAAG	Genotyping of *esd4-2* (*This study*)
